# Familial Pulmonary Alveolar Proteinosis and Type-I Interferonopathy by Mutation of *STAT2* (TIMS2)

**DOI:** 10.1084/jem.20251331

**Published:** 2026-07-16

**Authors:** Conor Gruber, Meredith Ramba, Bineeta Debnath, Lorenzo Cuollo, Anna-Lena Neehus, Angelica Lee, Sofija Buta, Marta Martin Fernandez, Jérémie Rosain, Laureline Berteloot, Philippe Drabent, Tom Le Voyer, Camille Soudée, Jessica Peel, Yoann Seeleuthner, Anne Puel, Shen-Ying Zhang, Michael Ciancanelli, Carlos A. Arango-Franco, Mélanie Migaud, Marie-Louise Frémond, Florence Renaldo, Odile Boespflug-Tanguy, Imen Dorboz, Beatrice Dubern, Tristan Fonteneau, Nima Parvaneh, Rasol Molatefi, Mohammad Shahrooei, Darragh Duffy, Vincent Bondet, Gillian I Rice, Yanick J Crow, Thierry Jo Molina, Nathalie Boddaert, Jean-Laurent Casanova, Veronique Houdouin, Isabelle Melki, Alice Hadchouel, Jacinta Bustamante, Dusan Bogunovic

**Affiliations:** 1 Department of Pediatrics, Vagelos College of Physicians and Surgeons, Columbia University Irving Medical Center, New York, NY, USA; 2 Columbia Center for Genetic Errors of Immunity, Vagelos College of Physicians and Surgeons, Columbia University, New York, NY, USA; 3 Division of Rheumatology, Children’s Hospital of Philadelphia, Pennsylvania, USA; 4 Imagine Institute, University of Paris Cité, INSERM U1163, Paris, France; 5 Laboratory of Human Genetics of Infectious Diseases, Necker Branch, INSERM U1163, Necker Hospital for Sick Children, Paris, France, EU; 6 Division of Hematology/Oncology, Boston Children’s Hospital and Department of Pediatric Oncology, Dana-Farber Cancer Institute, Harvard Medical School, Boston, MA, USA; 7 Rare Diseases Research Institute (IIER), Instituto de Salud Carlos III, Madrid, Spain; 8 Study Center for Primary Immunodeficiencies, Necker Hospital for Sick Children, Assistance Publique-Hôpitaux de Paris (AP-HP), Paris, France; 9 St. Giles Laboratory of Human Genetics of Infectious Diseases, Rockefeller Branch, Rockefeller University, New York, NY, USA; 10 Department of Pediatric Radiology, Necker Hospital for Sick Children, AP-HP, Paris, France; 11 Department of Pathology, Assistance Publique-Hôpitaux de Paris (AP-HP), Necker Hospital for Sick Children, Paris Cité University, Paris, France; 12 Department of Microbiology and Parasitology, School of Medicine, University of Antioquia, Medellín, Colombia; 13 Université Paris Cité, *Imagine* Institute, Laboratory of Neurogenetics and Neuroinflammation, INSERM UMR 1163, Paris, France; 14 Department of Paediatric Neurology, Armand-Trousseau Hospital, Assistance publique-Hôpitaux de Paris, Sorbonne University, Paris, France; 15 Centre de Référence Neurogénétique, Armand-Trousseau Hospital, Paris, France; 16 NeuroDiderot, Inserm U1141, Paris, France; 17 Department of Pediatric Neurology, Paris Cité University, Paris, France; 18 Pediatric Nutrition and Gastroenterology Department, Reference Center for Rare Disorders, PRADORT (Syndrome de PRADer-Willi et autres Obésités Rares avec Troubles du comportement alimentaire), Trousseau Hospital, AP-HP, Sorbonne University, Paris, France; 19 Service of Pediatric Pulmonology, RESPIRARE, Paris Cité University, Robert Debré Hospital, AP-HP, Paris, France; 20 Department of Pediatrics, Children’s Medical Center, Tehran University of Medical Sciences, Tehran, Iran; 21 Department of Pediatrics, Bo-Ali Children’s Hospital of Ardabil University of Medical Sciences, Ardabil, Iran; 22 Clinical and Diagnostic Immunology, KU Leuven, Leuven, Belgium; 23 Tehran University of Medical Sciences, Tehran, Iran; 24 Translational Immunology Unit, Institut Pasteur, Universite Paris Cite, Paris, France; 25 Division of Evolution, Infection and Genomic Sciences, School of Biological Sciences, Faculty of Biology, Medicine and Health, University of Manchester, Manchester, UK; 26 MRC Human Genetics Unit, Institute of Genetics and Cancer, University of Edinburgh, Edinburgh, UK; 27 Department of General Pediatrics, Pediatric Rheumatology Unit, Armand Trousseau Hospital, AP-HP, Sorbonne Université, FHU INFLAMME, Paris, France; 28 Department of Pediatric Pulmonology and Allergy, Necker Hospital for Sick Children, AP-HP, Paris, France; 29 INSERM U1151, Necker Institute, Paris, France & Paris Cité Université, Paris, France; 30 Division of Pediatric Allergy, Immunology and Rheumatology, Department of Pediatrics, Columbia University, New York, NY, USA

## Abstract

Mutations that enhance type I interferon (IFN-I) activity cause monogenic autoinflammatory disorders termed type I Interferonopathies. Along with the typical neurologic and rheumatologic manifestations, severe pulmonary disease is increasingly recognized yet poorly understood. We studied three siblings presenting with early-onset, life-threatening pulmonary alveolar proteinosis (PAP) and autoinflammatory stigmata. Genetic analysis uncovered a novel homozygous variant (R223Q) in STAT2, a key mediator of IFN-I signaling, which also facilitates feedback inhibition via USP18. R223Q STAT2 preserved signal transduction and viral control *in vitro*. However, cells homozygous for the R223Q variant failed to terminate IFN-I responses, owing to impaired localization of USP18. Unlike in classical forms of PAP, GM-CSF signaling remained intact. Instead, persistent IFN-I signaling antagonized monocyte migration toward chemokines essential for lung trafficking. Informed by these findings, the youngest sibling received JAK inhibitor and anti-IFN-I receptor therapy with marked clinical improvement. Collectively, Type-I Interferonopathy by Mutation of *STAT2* (TIMS2) compromises monocyte chemotaxis and underlies a novel mechanism of PAP.

## Introduction

Type-I interferons (IFN-I) are a family of highly-conserved and broadly-acting cytokines essential for antiviral immunity. The canonical IFN-I signaling pathway initiates as IFN-I binds to its cognate receptors, IFNAR1 and IFNAR2. The subsequent IFNAR heterodimerization allows for the receptor-associated kinases, JAK1 and TYK2, to phosphorylate STAT1 and STAT2, which then bind together with IRF9 to form a transcription factor complex, Interferon-Stimulated Gene Factor 3 (ISGF3). This complex induces transcription of hundreds of target genes, known collectively as Interferon-Stimulated Genes (ISGs). While these effectors orchestrate an intricately-regulated antimicrobial program under physiologic conditions, when dysregulated, the resulting clinical disease is devastating (Ivashkiv and Donlin, 2014, Crow and Casanova, 2024).

Both deficient and excessive IFN-I signaling underlies monogenic disease. Specifically, inborn errors of immunity (IEIs) that decrease the ISG response—which occur in deficiencies of viral sensing, IFN-I production or signal transduction—cause severe viral disease with variable penetrance. Among these are biallelic loss-of-function (LOF) variants in *STAT2* that reduce signal transduction (Alosaimi et al., 2019, Bucciol et al., 2023, Freij et al., 2020, Hambleton et al., 2013, Moens et al., 2017, Shahni et al., 2015). Such patients present in early childhood with severe viral infections, including critical pneumonias from respiratory syncytial virus (Lamborn et al., 2017), rhinovirus (Lamborn et al., 2017) or influenza (Ciancanelli et al., 2015).

In contrast, those Mendelian disorders that over-activate human IFN-I signaling lead to a group of autoinflammatory diseases termed type I interferonopathies (IFNopathies) (Crow and Stetson, 2022). These disorders arise from one of two mechanisms: increased production of IFN-I or enhanced responsiveness to IFN-I signal transduction (Crow and Stetson, 2022). Regarding the latter, all such disorders stem from loss of activity of USP18, a potent negative regulator that disassembles IFNAR dimerization and displaces JAK1 from the receptor (Malakhova et al., 2006, Wilmes et al., 2015). Loss of USP18 activity can occur via biallelic variants in the gene encoding for USP18 itself (Alsohime et al., 2020, Martin-Fernandez et al., 2022, Meuwissen et al., 2016), the stabilizing binding-partner ISG15 (Martin-Fernandez et al., 2020, Zhang et al., 2015), or the multifunctional intermediate STAT2 (Arimoto et al., 2017). This final genetic etiology was recently discovered to occur by rare variants that disrupt the secondary function of STAT2 to recruit USP18 to IFNAR, without affecting its primary pro-transcriptional role (Duncan et al., 2019, Gruber et al., 2020, Zhu et al., 2023).

The clinical presentation of IFNopathies is highly variable, with manifestations ranging from isolated asymptomatic intracranial calcifications to systemic inflammation, profound neurologic disease and life-threatening lung disease (Taft and Bogunovic, 2018). Classical pulmonary manifestations of etiologies related to increased IFN-I production (e.g. STING-associated vasculopathy with onset in infancy (SAVI) or Copa syndrome) include interstitial lung disease, diffuse alveolar hemorrhage and pulmonary hypertension (Cazzato et al., 2020). Distinct from these entities, pulmonary alveolar proteinosis (PAP) is characterized by accumulation of surfactant in the alveoli. The pathogenesis most often derives from alveolar macrophage dysfunction, typically due to genetic or acquired defects in GM-CSF signaling. While PAP was recently linked with IFNopathy by *STAT2* variants (Gruber et al., 2020), the mechanistic underpinnings remain unknown. Herein, we investigate three siblings harboring a novel biallelic variant in STAT2 (R223Q) that presented with early-onset PAP.

## Methods

### Extended Case Report

**P1** was born at 38 weeks gestation without complication. At age 4 months, she presented with cough and increased work of breathing, which progressed to recurrent episodes of respiratory failure. Work-up revealed bilateral diffuse patchy and ground-glass opacities with a “crazy paving” appearance on chest computerized tomography (CT). Bronchoalveolar lavage (BAL) was performed, with fluid studies consistent with a diagnosis of pulmonary alveolar proteinosis (PAP)(Table 1). A complete etiologic work-up for PAP was unrevealing other than positive serologies for anti-nuclear antibodies (ANA), anti-smooth muscle antibodies and anti-thyroglobulin antibodies. In addition, the following extra-pulmonary features were observed: hepatomegaly with elevated transaminase levels; recurrent episodes of unexplained hypoglycemia; episodes of seizure-like activity with abnormal electroencephalogram correlates starting at 6 months of age; and meningoencephalitis at 13 months of age, as evidence by high protein level in the cerebrospinal fluid (CSF) and signal abnormalities on brain MRI.

Given the PAP diagnosis, several whole lung lavage (WLL) therapies (approximately 1-2 per month) were performed and supplemental oxygen support was provided. At 9 months of age, she developed a pneumonia due to *Pseudomonas aeruginosa* and was treated with Ceftazidime and corticosteroids. At 11 months of age, she was admitted to the intensive care unit (ICU) for respiratory failure. Bacterial cultures and viral testing were negative. Between 12 to 16 months of age, she was hospitalized for multiple respiratory decompensations, with the identification of different microbial agents, such as adenovirus, parainfluenza, and *P. aeruginosa*. At 17 months, she suffered severe respiratory failure requiring mechanical ventilation. She received bolus corticosteroids, cyclophosphamide and WLL. A lung biopsy detected *Mycobacterium xenopi*. Rifampin, clarithromycin and ethambutol were added as treatment. However, she died at age 19 months.

**P2** was born at 37 weeks gestation without complication. At 3 months of age, she presented with dyspnea. Physical exam was notable for respiratory distress and hepatomegaly. Testing was positive for respiratory syncytial virus (RSV). She required increased supplemental oxygen and was ultimately transferred to the ICU for mechanical ventilation. Chest radiography showed a bilateral interstitial pattern. Chest CT demonstrated interstitial and alveolar infiltration with a characteristic “crazy-paving” appearance in which interlobular and intralobular septal thickening are superimposed on ground-glass opacities. Given these findings suspicious for PAP, an initial WLL was attempted but incompletely tolerated. The lavage fluid obtained was milky in color, consistent with PAP (Table 1). Etiologic work-up and extra-pulmonary investigations revealed only weakly-positive ANA titers (1:1000) and elevated transaminase levels, respectively. No brain imaging was performed. No hypoglycemic issues were noted. A second WLL was undertaken on the left side, for which testing was negative for bacteria, acid-fast bacilli, viruses and fungi (including *Pneumocystis jirovecii)*. HIV testing was negative. She required stress-dose corticosteroids and inotropic support. Unfortunately, she died of respiratory failure 15 days after her admission to the ICU.

**P3** was born at 40 weeks gestation without complication. At one month of age, he had an auricular right adenomegaly requiring surgical excision. Due to the aforementioned family history of respiratory failure in his siblings, a CT of his chest was performed, which revealed a pattern suggestive of interstitial lung disease. At 4 months of age, a BAL was performed and a diagnosis of PAP was established. At age 5 months, he developed acute aseptic meningoencephalitis. CSF studies demonstrated lymphocytic pleocytosis with mild protein elevation, neuroinflammatory marker elevation (neopterin and biopterins) and an absence of oligoclonal bands. At the time, interferon antiviral activity was negative in blood and CSF (<2UI). ANA and anti-double strand DNA antibodies were negative at this evaluation. The patient was treated successfully with parenteral corticosteroids.

At 6 months of age, he developed respiratory distress in the setting of positive rhinovirus / enterovirus testing. He was also noted to have premature obesity with polyphagia. At 2 years-old, he was hospitalized for hypoxic respiratory failure due to a suspected pulmonary infection; however, all microbial testing was negative. At 3 years-old, he was admitted to the ICU with another documented rhinovirus infection. At the time, a CT scan demonstrated multiple bilateral opacities and PAP. He required mechanical ventilation and WLL therapy. Subsequently, he developed another respiratory decompensation with positive Parainfluenza pneumonia. At 5 years-old, he was again hospitalized due to respiratory issues, with radiologic evidence of a PAP exacerbation. He again required WLL treatment. A head CT scan was performed showing multiple cerebral calcifications. Subsequent MRI demonstrated the intra-axial, infra-tentorial and supra-tentorial calcified lesions, with punctiform calcifications in the bulbo-pontine, basal ganglia and subcortical regions. In parallel, ongoing ANA positivity (1/1280) was detected, although with negative testing for extractable nuclear antigen autoantibodies. Further studies demonstrated antineutrophil cytoplasmic antibodies (cANCA, 1/320 titer) and negative anti-cyclic citrullinated peptide antibody serologies. By immunophenotyping, all lymphocyte subsets were present at or near normal frequencies. (Table 2).

### Whole-exome and Sanger Sequencing

Whole exome sequencing (WES) was performed from genomic DNA extracted from PMBCs in P1, P2 and their parents by IntegraGen SA (Evry, France). WES for P3 was performed on genomic DNA extracted from whole blood. DNA was captured using Agilent in-solution enrichment methodology (SureSelect SureSelect XT Clinical Research Exome, Agilent) with the biotinylated oligonucleotides probes library (SureSelect XT Clinical Reasearch Exome - 54 Mb, Agilent), followed by paired-end 75 bp sequencing on an Illumina HiSeq4000. WES results were analyzed by filtering for homozygous variants with a Combined Annotation Dependent Deletion score (CADD) above the gene-specific mutation specific cutoff, a GDI below 13.41, and a minor allele frequency of <0.01 in gnomAD. For confirmation of the *STAT2* variant and familial segregation patterns, STAT2 was amplified from DNA by PCR using gene-specific primers and the DreamTaq Polymerase (Thermo Fisher Scientific). Sanger sequencing was performed with the BigDye Terminator v.3.1 sequencing kit (Thermo Fisher Scientific) and analysis was done using ABI Prism 3700 (Applied Biosystems). For STAT2 nucleotide and amino acid positions, the following NCBI transcript and protein accession numbers were used, respectively: NM_005419.4 and NP_005410.1.

### Cytokine Analysis

BAL samples were analyzed for the presence of inflammatory cytokines and chemokines with the following LEGENDplex™ assays (all from BioLegend): Human Inflammation Panel 1 (#740809, BioLegend), Human Inflammation Panel 2 (#740883), Human Proinflammatory Chemokine Panel 1 (#740985) and Human Proinflammatory Chemokine Panel 2 (#741183) according to the manufacturer’s instructions. Samples were analyzed by flow cytometry on a Gallios flow cytometer. Data analysis was performed with LEGENDplex Cloud-based Data Analysis Software (BioLegend). All plasma and BAL samples were obtained from patients and controls at infection-free time points (culture and PCR negative). IFN-α was quantified at the protein level in serum and BAL samples using the Pan-IFN-α assay developed for digital-ELISA Simoa technology, as previously described (Rodero et al., 2017).

### Cell Culture

Dermal primary fibroblasts were obtained by punch biopsy from patients or controls, and immortalized by transduction of Human Telomerase Reverse Transcriptase (hTERT). EBV-immortalized B-cell lymphoblast lines were generated by infection of PBMCs derived from peripheral blood of patients or controls. The U6A cell line was gifted by Dr. Sandra Pellegrini. THP-1 cells were obtained from the ATCC (RRID:CVCL_0006). Adherent and suspension lines were cultured in DMEM (Gibco) or RPMI (Gibco), respectively. Media were supplemented with 10% FBS (Invitrogen), GlutaMAX (350ng/ml; Gibco) and 1% penicillin/ streptomycin (Gibco). All cells were cultured in incubators at 37°C and 5% CO_2_. All cell lines were routinely tested for mycoplasma contamination with the e-Myco PCR Mycoplasma Detection Kit (Fisher Scientific) according to the manufacturer’s instructions. For experimentation, adherent cell lines were plated in tissue-culture treated plates and stimulated with the indicated concentrations of IFN-α2b (Intron A, Merck) prepared in complete DMEM media. Suspension cells were maintained in non-tissue-culture treated vessels for experimentation and culturing.

### RNA Expression

For *in vitro* studies, RNA was extracted from U6A cells, hTERT-immortalized fibroblasts (Qiagen RNeasy) or whole blood (PAXgene Blood RNA Kit). cDNA was generated by reverse-transcription (High-Capacity cDNA Reverse Transcription Kit). The expression of ISGs (*IFI27, IFIT1, MX1, USP18, RSAD2*, and *ISG15*), relative to *GAPDH* housekeeper gene, was analyzed by TaqMan quantitative real-time PCR (TaqMan Universal Master Mix II with UNG) on QuantStudio 6 Pro Real-Time PCR System (Thermo Fisher Scientific). Relative expression was derived by the delta-delta Ct method.

To assess ISG expression in whole blood, the NanoString platform was used, as previously described (Lepelley et al., 2021). In brief, total RNA was extracted from whole blood using the PAXgene (PreAnalytix) RNA isolation kit. The Agilent TapeStation system (RRID:SCR_019547) was used to assess the quality of the RNA. For each sample, 100 ng total RNA was used. A panel of 24 ISG probes (*IFI27, IFI44L, IFIT1, ISG15, RSAD2, SIGLEC1, CMPK2, DDX60, EPSTI1, FBXO39, HERC5, HES4, IFI44, IFI6, IFIH1, IRF7, LAMP3, LY6E, MX1, NRIR, OAS1, OASL, OTOF, and SPATS2L*) and 3 housekeeping genes (NRDC, OTUD5, and TUBB) was generated according to the manufacturer’s recommendations. All data were analyzed using the nSolver Software (NanoString Technologies). Expression values were normalized relative to the internal positive and negative calibrators, the three housekeeping genes, and the control samples. The median expression value for each ISG was calculated across healthy control samples. A threshold for positivity was defined as a NanoString score equal to two standard deviations above the mean healthy control value.

### Knockout Cell Lines

*CCR2-/-* THP-1 cells were generated as previously described (Neehus et al., 2023). *USP18* and *STAT2* were knocked out in a similar fashion using CRISPR-Cas9 ribonucleoprotein complexes (IDT), as per the manufacturer’s instructions. In brief, sgRNA and Cas9 (Alt-R™ S.p. Cas9 Nuclease V3, IDT) were transfected into THP-1 cells by nucleofection using the SG nucleofection kit (Lonza) with Alt-R CRISPR-Cas9 Electroporation Enhancer (IDT) on a 4D-Nucleofector System (Lonza, Pulse Code FF-100). Cells were allowed to recover and proliferate for one week before single-cell plating and clonal expansion. Success of gene knock-out was validated by western blotting.

### Ectopic Expression

The R223Q and R148Q variants were generated by site-directed mutagenesis using QuickChange II PCR (Agilent) on a WT *STAT2* lentiviral-compatible plasmid (Gruber et al., 2020). An analogous *Luciferase* expressing vector was used for a null control. For lentivirus generation, HEK293T cells (RRID:CVCL_0063) were first transfected with *STAT2, pMD2*, and *psPAX2* by CaCl2 transfection. Forty-eight hours later, the supernatants were collected, which were then used to transduce U6A cells or THP-1 cells with polybrene. The cells were selected with puromycin treatment (0.4 µg/ml). The genes used for ectopic expression and co-immunoprecipitation were pTRIP-*USP18*-V5, pMET7-*IFNAR2*, and pTRIP-*STAT2* (WT, R223Q, and R148Q) (Gruber et al., 2020).

### Immunoblotting

After the indicated cytokine stimulation, cells were washed of cytokine, and lysed by incubation in Radio-Immunoprecipitation Assay lysis buffer (Thermo Fisher Scientific) with 1x Protease/Phosphatase inhibitor cocktail (Cell Signaling Technology) for 10 minutes on ice. The samples were centrifuged at 16,000g for 10 minutes. Subsequently, the insoluble components were removed. Next, the samples were boiled at 95 degrees Celsius for 5 minutes with NuPAGE LDS Sample buffer (Thermo Fisher) and dithiothreitol. The samples then underwent gel electrophoresis and transferred to a PVDF membrane using the Bio-Rad Western blot workflow. The membranes were blocked in 5% BSA with primary antibodies overnight at 4°C, followed by secondary antibody incubation in 5% nonfat dry milk at room temperature for 1 hour. Antibodies used were against STAT2 (Cell Signaling Technology, AB_2799824), phospho-Tyr701 STAT1 (Cell Signaling Technology, AB_3679336), phospho-Tyr 689 STAT2 (Cell Signaling Technology AB_2800123), phospho-STAT5 (Cell Signaling, AB_823649), USP18 (Cell Signaling Technology, AB_10614342), β-actin (Cell Signaling Technology, AB_2242334), GAPDH (Cell Signaling Technology, AB_2756824), phospho-AKT (Cell Signaling Technology, AB_329825), phospho-ERK (Cell Signaling) and FLAG (M2, Sigma, AB_262044). Signal was detected with enhanced chemiluminescence detection reagent, SuperSignal West Pico (Thermo Fisher Scientific) and SuperSignal West Femto (Thermo Fisher Scientific) by imaging with the Amersham ImageQuant 800 Western blot imaging system (Cytiva).

### Co-Immunoprecipitation Assay

U6A cells were plated in tissue-culture-treated plates. The following day, cells were transiently transfected with pTRIP-USP18-V5, IFNAR2-Flag, pTRIP-STAT2 (WT, R223Q, and R148Q), and pTRIP-Luciferase by Lipofectamine 2000 (Thermo Fisher Scientific) or *Trans*It-X2 Dynamic Delivery System (Mirus) according to the manufacturer’s instructions. After 24 hours, cells were lysed in 50 mM Tris, pH 6.8, 0.5% NP-40, 200 mM NaCl, 10% glycerol, 1 mM EDTA, and 1× Protease/Phosphatase inhibitor cocktail (Cell Signaling Technology) and collected using the cell scrapers. Then the lysates were incubated with protein G dynabeads (Thermo Fisher Scientific) or anti-flag M2 magnetic beads (Sigma-Adlrich) overnight at 4°C. The following day, cell lysates incubated with protein G dynabeads were mixed with V5 antibody for 20 minutes at room temperature. Immunoprecipitates were eluted and then subjected to Western blotting.

### Viral Infection

Primary fibroblasts were plated at a density of 2 × 10^4^ cells per well in 96-well plates, in DMEM with or without IFNα2b (10^5^ IU/mL, Schering-Plough) treatment for 18 hours as previously described (Gao et al., 2021). The medium was replaced with medium containing VSV at the indicated MOIs. Mortality was assessed 24 hours after infection, with the LDH Cytoxicity Detection Kit^PLUS^ (Roche). The death of cells is expressed relative to that of uninfected cells. Viability was assessed in a resazurin-based viability assay (MilliporeSigma). The viability of infected cells is expressed relative to that of uninfected cells.

### Serum Anti-Cytokine Levels and GM-CSF Neutralization

Plasma samples were obtained from whole blood of healthy individuals, patients with neutralizing autoantibodies to GM-CSF and P3; and stored in aliquots at -80°C until testing. Human peripheral blood mononuclear cells (PBMCs) from healthy controls were isolated from whole blood by Ficoll-Hypaque density centrifugation (Amersham Pharmacia Biotech, Sweden). PBMCs were counted and plated at 5 × 10^5^/well in 96 V-bottom plates (Thermo Fisher Scientific) in 100 µL of RPMI (Gibco BRL, Invitrogen), supplemented with 10% fetal bovine serum (FBS) (Gibco BRL, Invitrogen) or 100 µL of RPMI supplemented with 10% plasma from patients or controls. PBMCs were left unstimulated or stimulated with 5 or 20 ng/mL rhGM-CSF or 100 ng/mL of rhIL-3 (Miltenyi Biotec) for 15 minutes at 37°C. Then, cells were fixed and permeabilized with a fixation/permeabilization kit (eBioscience). Extracellular labeling was performed with CD14-Pacific Blue and CD4-FITC (Sony Biotechnology, clones M5E2 and RPA-T4, respectively). Cell viability was determined with the Aqua Dead Cell Stain Kit (Thermo Fisher Scientific) and phosphorylation of STAT5 (p-STAT5) by intracellular staining with Phospho- Flow PE Mouse Anti-p-STAT5 (pY694) antibody (BD Biosciences, AB_399858). Data were collected using a Gallios flow cytometer (Beckman Coulter) and analyzed with the FlowJo software v.10.6.2 (Becton Dickinson, RRID:SCR_008520).

The remainder of anti-cytokine antibodies were measured by a flow cytometry-based assay. In brief, patient or control sera was diluted and incubated with a 23-panel array of beads coupled to the following cytokines: GM-CSF, IFN-α, IFN-β, IFN-γ, IFN-ω, IL-6, IL-12, IL-17A, IL-17F, IL-22, IL-23, IL-27, TNF, TGF-β, IL-3, IL-4, FLT3L, BAFF, CCL-2/MCP-1, IL-1β, IL-21, IL-10, IL-13, IL-15, IL-1α, IL-5, IL-8, IL-9, M-CSF, TSLP, CXCL1, TNF-β, IL-17E. Beads were then incubated with a PE-conjugated anti-human IgG secondary antibody. Samples were then analyzed by flow cytometry to detect for IgG reactivity to cytokine-coupled beads.

### Transwell Migration

Prior to assaying chemotaxis, THP-1 cells were incubated at the indicated concentrations and incubation periods of IFN-α (Intron A, Merck). For pharmacologic treatments, cells were incubated with either drug baricitinib (MedChem Express) or anifrolumab (MedChem Express) prior to IFN-α stimulation. As negative controls, DMSO or human IgG isotype antibodies (Jackson Immuno Research Lab) were used at an equivalent dose to the highest corresponding drug treatment. A 96-well permeable trans-well system containing 5.0 µm pore polycarbonate inserts (Corning) was then preloaded in the lower chamber with media containing chemokine and flow cytometry counting beads. Chemokines were used at the following concentrations: CCL-2 20 ng/ml (BioLegend), CCL-3 20 ng/ml (BioLegend), CCL-5 500 ng/ml (BioLegend), CXCL-12 (20 ng/ml). To perform the migration assay, cells were loaded onto the top chamber and allowed to migrate in a cell culture incubator for 2 hours. The contents of the bottom chamber were then analyzed for cell and bead counts on a flow cytometer (Novocyte, Agilent), for which an equal volume was acquired for each sample. Migration was then calculated as the ratio of cells to beads, and when indicated, normalized relative to the control condition (CCL2+, unstimulated with IFN-I).

### CCR2 Surface Expression

THP-1 cells were pre-treated with interferon alpha (IFN-α) for 100 IU/mL for 8 hours, washed with PBS, and then allowed to rest for 36 hours in complete media. Cells were then harvested and processed for flow cytometry. In brief, cells were stained with Live/Dead Fixable Viability Dye (Biolegend) and labeled with anti-CCR2 antibody (Biolegend clone K036C2, AB_2562058). A species-matched isotype control antibody was used as a comparator. Labeled cells were then processed on a flow cytometer (Novocyte, Agilent) to quantify the median signal intensity of live cells.

### Study Approval

All study participants provided written informed consent for their samples to be used as part of this research project. The study was reviewed and approved by the French Ethics Committee “Comité de Protection des Personnes” the French National Agency for Medicine and Health Product Safety and the “Institut National de la Santé et de la Recherche Médicale” in France (protocol 2022-A00257-36), as well as the Rockefeller University Institutional Review Board in New York (protocol JCA-0699).

### Online Supplemental Material

Figure S1 shows additional views and timepoints of the CT chest imaging from P1-P3, which was consistent with the radiologic findings in PAP. Figure S2 provides further brain imaging by MRI of P1 and P3. Figure S3 shows the Sanger sequencing performed to validate the *STAT2* genotype across members of the family under study. Figure S4 shows STAT2 expression and proximal IFN-I signaling responses in EBV-immortalized B-cells derived from P3 and a healthy donor. Figure S5, relating to Figure 5, shows the lung immunohistochemical and peripheral blood cytokine patterns in STAT2 R223Q patients, as well as in vitro experiments on GM-CSF and AKT/ERK signaling.

## Results

### Familial PAP and recurrent infections

**Figure 1:**
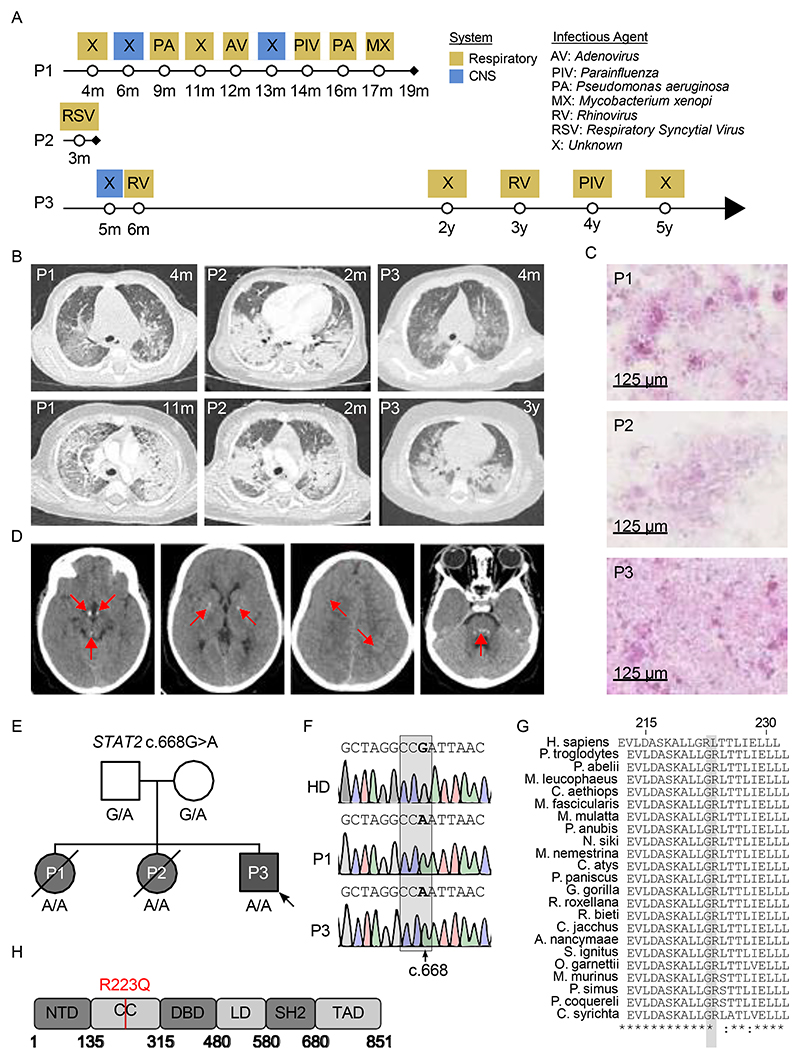
A homozygous *STAT2* variant identified in siblings with infection-associated recurrent respiratory failure. (A) Clinical timeline demonstrating respiratory (gold) and neurologic (blue) decompensations in association with apparent infections (abbreviations defined in the panel). (B) Chest CT scans showing characteristic findings of PAP, including ground-glass opacities and thickening of interlobular and intralobular septal lines. (C) Cytological analysis of BAL fluid demonstrating extracellular lipoproteinaceous material present in association with macrophages for all the patients, as indicated by positive staining with Periodic acid-Schiff (PAS). Scale bars indicate 125 micrometers. (D) CT imaging of the brain from P3 demonstrating intracranial calcifications (red arrows) involving the brainstem, periventricular regions, basal ganglia and subcortical zones (left to right). (E) Family pedigree and c.668G>A genotype at the *STAT2* locus. (F) Sanger sequencing from dermal fibroblasts derived from a healthy donor, P1 and P3. Data representative of two independent experiments. (G) Amino acid alignment of STAT2 orthologs across primate species. (H) Location of the R223Q substitution (red) in the linear structure of STAT2 and its domains, namely the N-terminal domain (NTD), coiled-coil domain (CC), DNA-binding domain (DBD), linker domain (LD), Src homology domain (SH2) and transactivation domain (TAD).

Here, we studied three siblings (P1-P3) from a nonconsanguineous family originating from Cape Verde and living in France. All patients were born at term without complication but later developed respiratory symptoms within the first 6 months of life (Table 1). P1 first presented at age 4 months with a presumed interstitial pneumonia, albeit with negative infectious studies. After this initial episode, she developed recurrent pulmonary disease exacerbations requiring respiratory support, some of which were associated with the presence of infectious agents, including parainfluenza, adenovirus, *Pseudomonas aeruginosa* and *Mycobacterium xenopi* (Figure 1A). At the age of 19 months, she died from respiratory failure. P2 suffered a more rapidly progressive course. She developed respiratory failure in the setting of an RSV infection at 3 months of age requiring mechanical ventilation and leading to death (Figure 1A). Finally, P3 first presented at 5 months of age with aseptic meningoencephalitis. Shortly after, he developed a respiratory decompensation for which rhinovirus was detected. At the initiation of this study, P3 was 5 years old and had been hospitalized for at least 5 respiratory decompensations, which were variably associated with the detection of viruses (Figure 1A).

In each case, chest CT imaging was performed and demonstrated ground-glass opacities superimposed with interlobular septal thickening and intralobular lines forming the so-called “crazy-paving” pattern suggestive of PAP (Figure 1B, Supplementary Figure 1). BAL returned milky-opaque fluid consistent with PAP. Specifically, cytopathology demonstrated large foamy macrophages engorged with PAS–positive and Oil Red-O positive inclusions and extracellular granular material, equally PAS-positive (Table 1, Figure 1C). Given this diagnosis, several whole lung lavage (WLL) therapies were performed. The remainder of the clinical work-up was significant for hepatomegaly with elevated transaminases (P1-P2); lymphadenopathy requiring surgery (P3); variable presence of auto-antibodies (P1-P3) (Table 1); and intracranial calcifications on head CT (P3; not assessed in P1 & P2) (Figure 1D) and periventricular white matter lesions by cranial MRI (P1 & P3) (Supplementary Figure 2).

### A homozygous missense variant in the coiled-coil domain of STAT2

We performed WES in P1, P2 and P3 to test the hypothesis that a monogenic disorder underlies clinical disease. The homozygosity rates for P1, P2 and P3 were 0.595%, 0.785% and 0.668%, respectively, confirming non-consanguinity. Given the early presentation and absence of disease in the parents (Figure 1E), genetic analysis focused on autosomal recessive inheritance arising from rare (i.e., minor allele frequency (MAF) < 0.01 in gnomAD v.4.1.1), non-synonymous variants predicted to be deleterious. This analysis revealed a biallelic single-nucleotide substitution c.668G>A in the *STAT2* gene. Sanger sequencing of all patients and their parents confirmed autosomal recessive inheritance (Figure 1E-F, Supplementary Figure 3). No other genetic variants that segregated with disease in the family were predicted to be disease-causing, including those underlying IEIs or monogenic PAP. While the c.668G>A *STAT2* variant is present among publicly-available genomes, it is exceedingly rare (MAF 8.05E-6 in the gnomAD database) and no homozygotes have been reported.

This substitution generated a missense variant, p.R223Q, in a residue universally-conserved among primates (Figure 1G). In the linear structure of STAT2, this residue localizes to exon 8 of the coiled-coil domain (CCD) that binds IRF9, which is essential for ISGF3 signaling (Figure 1H). Initially discovered by Arimoto et al, and later projected *in silico* by Zhu et al, these CCD residues are also key for USP18 binding and negative regulation (Zhu et al., 2023, Arimoto et al., 2017). Potentially reflecting the complex functional nature of this domain, pathogenicity prediction programs indicated divergent results on the deleteriousness of the R223Q variant (Table 3).

### Intact IFN-I signaling and adequate antiviral responses with R223Q STAT2

Given the close association between clinical exacerbations and the detection of infectious agents, we hypothesized that disease pathogenesis could originate from either (1) hypomorphic IFN-I signaling leading to increased infectious susceptibility or (2) overexuberant IFN-I signaling triggered by immune stimulation.

**Figure 2:**
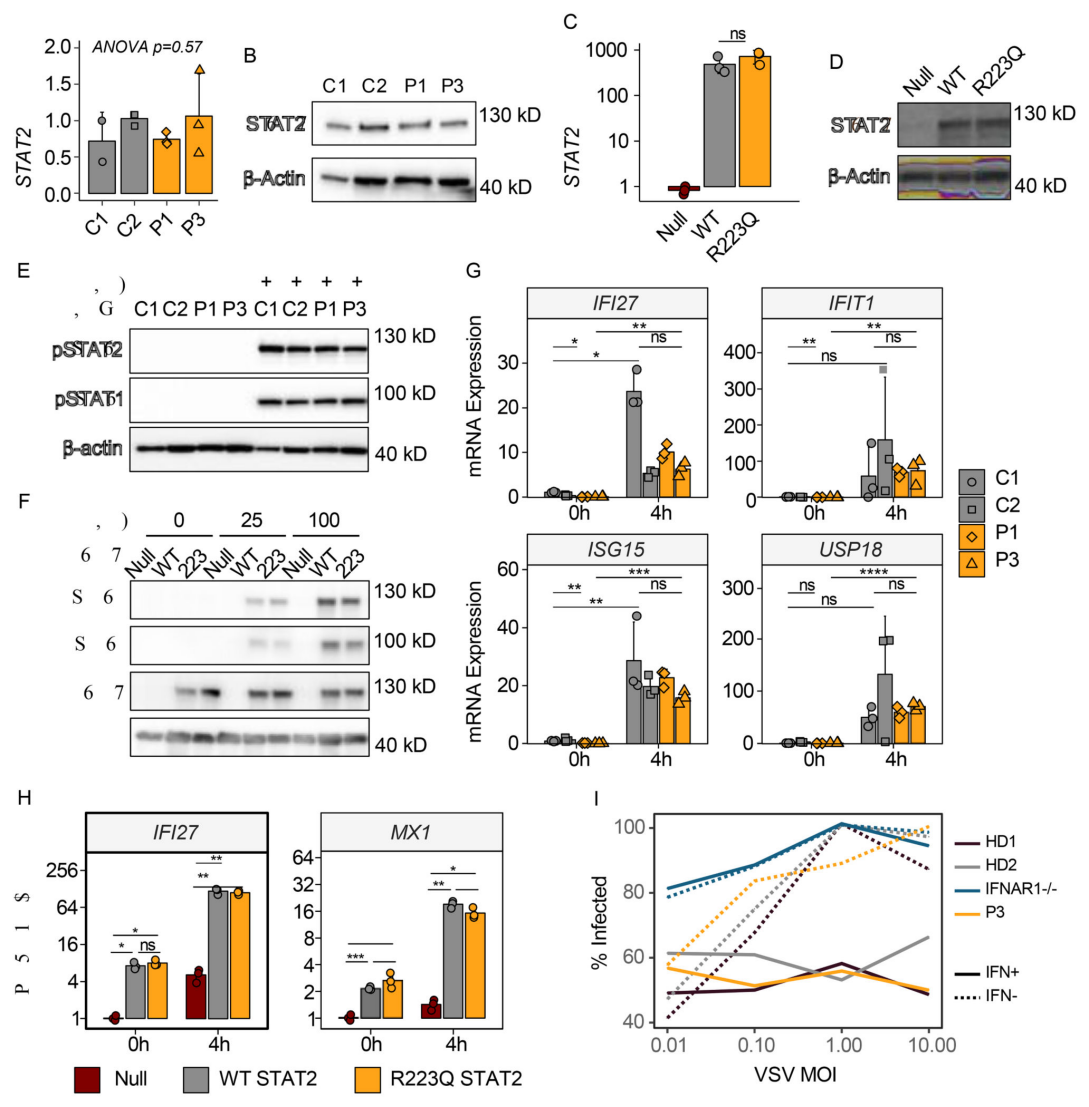
The R223Q STAT2 variant preserves normal expression, signal transduction and antiviral immunity. (A) *STAT2* mRNA expression by qPCR from hTERT-immortalized dermal fibroblasts from healthy donors (C1 and C2, indicated in gray) and patients (P1 and P3, indicated in yellow). Bars indicate mean ± SEM. Individual points represent the mean of technical triplicates for each biological replicate (n=2-3), as indicated in the figure. Results of one-way ANOVA testing reported. (B) Protein expression by immunoblotting for STAT2 in hTERT-immortalized dermal fibroblasts. (C) *STAT2* mRNA expression by qPCR from U6A *STAT2*-/- cells transduced with an empty vector (red), WT *STAT2* (gray) or variant *STAT2* encoding R223Q (yellow). Bars indicate mean ± SEM. Individual points represent the mean of technical triplicates for each biological replicate (n=3), as indicated in the figure. (D) Protein expression by immunoblotting in STAT2-reconstituted U6A cells. (E) STAT phosphorylation by immunoblotting in dermal fibroblasts after a 15-minute stimulation with 100 IU/mL of IFN-I. (F) STAT phosphorylation by immunoblotting in STAT2-reconstituted U6A cells after a 15-minute stimulation with the indicated doses of IFN-I (IU/mL). (G) qPCR for ISG expression in fibroblasts from healthy donors (gray) and patients (yellow) after 4 hours of IFN-I stimulation (100 IU/mL). For statistical testing, individual subjects were combined into their respective patient groups: healthy donor [C1 and C2] or patient [P1 and P3]. Bars indicate mean ± SEM. Individual points represent the mean of technical triplicates for each biological replicate (n=3), as indicated in the figure. (H) qPCR for ISG expression in U6A *STAT2*-/- cells transduced with an empty vector (red), WT *STAT2* (gray) or variant *STAT2* encoding R223Q (yellow) after 4 hours of IFN-I stimulation (100 IU/mL). (I) VSV infection in IFN-stimulated (dotted line) or unstimulated (solid line) fibroblasts from healthy donors (black/gray), P3 (yellow), or an individual with IFNAR1 deficiency (blue). Points represent mean values of n=3 replicates. Unless otherwise indicated, statistical testing was performed with unpaired t-tests with Holm’s correction for multiple comparisons, when applicable. P-values are abbreviated *P < 0.05, **P < 0.01, ***P < 0.001, or ns = not significant P > 0.05. Representative results of at least 3 independent experiments are shown for each panel.

To begin interrogating the consequences of this variant, we first assayed its effects on STAT2 quantity, as IFN-I-enhancing mutations require intact protein production, whereas all loss-of-function variants discovered to date disrupt protein expression. In hTERT fibroblasts from P1 and P3, both mRNA and protein levels (Figure 2A & B) were similar to cells from healthy donors. Next, WT and R223Q STAT2 were cloned into a lentiviral vector and transduced into a STAT2-deficient cell line, U6A (Pellegrini et al., 1989). Similarly, mRNA and protein for the R223Q variant was expressed in a WT-like fashion (Figure 2C & D).

Knowing that R223Q does not affect STAT2 quantity, we next sought to study the downstream effects on the signaling pathway. When briefly stimulated (15 minutes) with IFN-I, both healthy and patient cells demonstrated a similar capacity to phosphorylate STAT2 and its binding partner STAT1 (Figure 2E). These results were confirmed in the isogenic system using WT and R223Q STAT2-reconstituted U6A cells (Figure 2F). When target gene expression was measured after 4 hours of IFN-I stimulation, induction of ISGs in the patient fibroblasts was similar to that of healthy donor fibroblasts (Figure 2G). Likewise, U6A cells demonstrated similar levels of ISG induction whether reconstituted with WT or R223Q STAT2 (Figure 2H). Accordingly, these intact IFN-I responses correlated with normal antiviral function, as patient and healthy donor fibroblasts exhibited comparable rates of infection with Vesicular Stomatitis Virus (VSV), in contrast to the heightened susceptibility of IFNAR1-/- cells (Figure 2I)

### STAT2 R223Q enhances ISG expression at late timepoints

**Figure 3:**
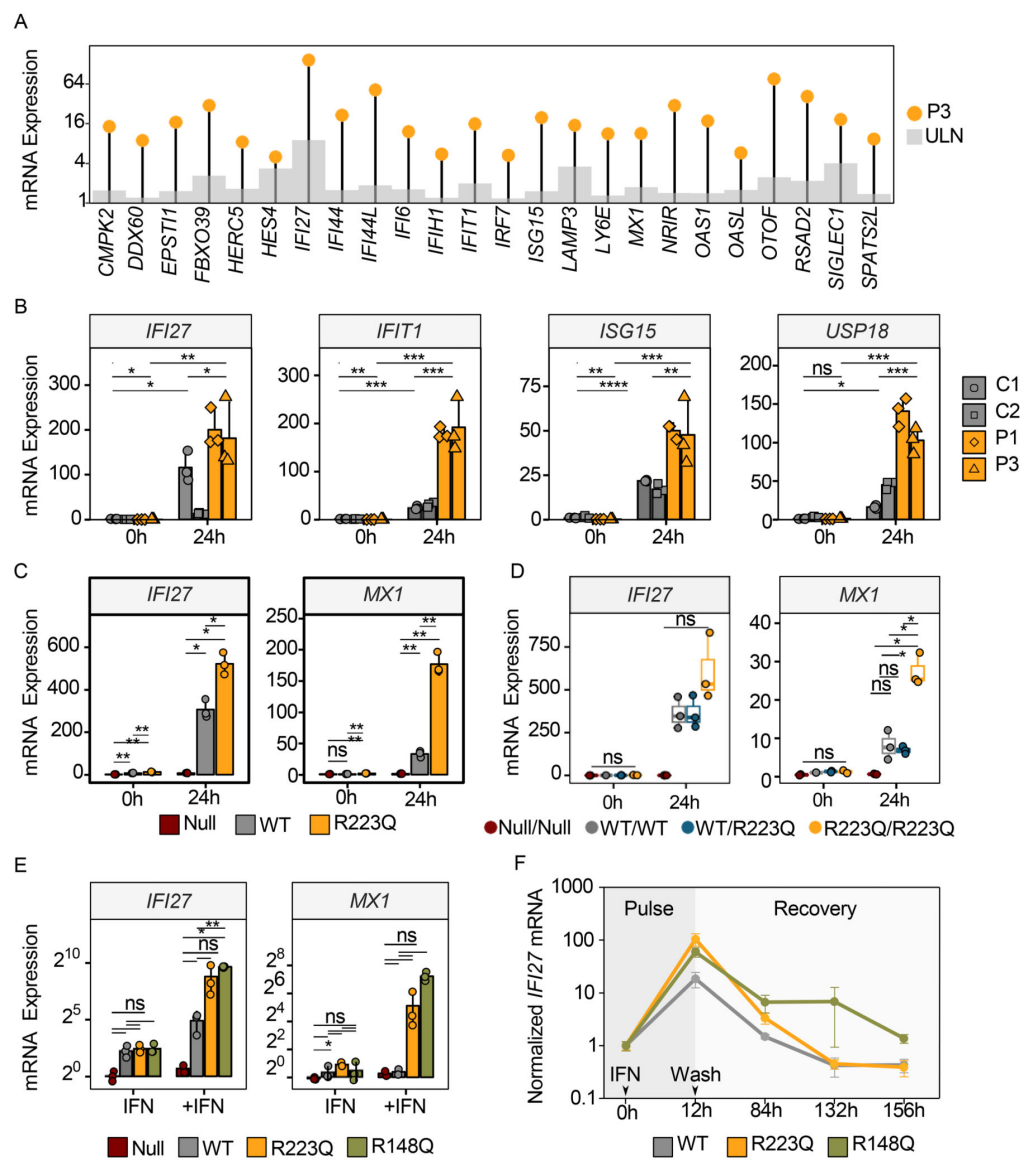
Chronic IFN stimulation leads to persistent ISG expression *in vivo* and *in vitro*. (A) NanoString assay for quantification of ISG expression in peripheral blood obtained from P3 (yellow, n=1) and healthy controls (gray, represented as the upper limit of normal (ULN) of the 95% confidence interval). Data represents a single experiment. (B) qPCR for ISG expression in hTERT-immortalized dermal fibroblasts from healthy donors (gray; C1 and C2) and patients (P1 and P3, yellow) stimulated with IFN-I (100 IU/mL) for 24 hours. For statistical testing, individual subjects were combined into respective groups: healthy donor [C1 and C2] or patient [P1 and P3]. (C) qPCR for ISG expression in U6A *STAT2*-/- cells transduced with an empty vector (red), WT *STAT2* (gray) or variant *STAT2* encoding R223Q (yellow). Cells were stimulated with IFN-I (100 IU/mL) for 24 hours. (D) qPCR for ISG expression in a dual-transduction system of U6A *STAT2*-/- reconstituted with equivalent levels of empty vector, WT *STAT2* (gray), variant *STAT2* encoding R223Q (yellow), or an equal quantity of WT and variant STAT2 (blue). Cells were stimulated with IFN-I (100 IU/mL) for 24 hours. (E) qPCR for ISG expression after 24-hour stimulation (100 IU/mL) with IFN-I in U6A *STAT2*-/- cells transduced with an empty vector (red), WT *STAT2* (gray), variant *STAT2* encoding R223Q (yellow) or R148Q (green). (F) qPCR for ISG resolution in dermal fibroblasts stimulated for 12 hours with IFN-I (10 IU/mL) and sampled at the indicated timepoints. Data is shown for a representative ISG, *IFI27*, and expression is normalized by cell type to the unstimulated condition. Statistical testing was performed with unpaired t-tests with Holm’s correction for multiple comparisons, when applicable. P-values are abbreviated *P < 0.05, **P < 0.01, ***P < 0.001, or ns = not significant P > 0.05. Bars indicate mean ± SEM. Unless otherwise indicated, data are representative of three independent experiments performed with technical triplicates of biological replicates (n=3).

As IFN-I signaling was sufficient for antiviral effector function in patient cells, we then tested whether overexuberant responses to immune stimuli were instigating pathogenic inflammation. To assess this hypothesis, we first analyzed ISG levels in the patient’s peripheral whole blood (only available in P3). A marked upregulation in the mRNA expression of 24 ISGs was observed in P3 as compared to healthy donors (Figure 3A). In accordance, elevated levels of IFNα cytokine were detected in the serum of P3 (51.3 fg/ml; normal range <10 fg/ml) by digital ELISA (Rodero et al., 2017).

We reasoned that the marked elevation of ISGs might result from the prolonged kinetics of immune stimulation that can be experienced *in vivo*. To assess this possibility, we exposed cells *in vitro* to prolonged periods of IFN-I (24 hours). At these late timepoints, patient fibroblasts expressed higher levels of ISGs relative to healthy donor fibroblasts (Figure 3B). These findings were also redemonstrated in STAT2-reconstituted U6A cells (Figure 3C). This isogenic system substantiates the R223Q STAT2 variant as sufficient to increase IFN-I responsiveness.

Next, we tested whether homozygosity was required for the hyperactive ISG signaling phenotype. To accomplish this, we transduced U6A cells with STAT2 lentiviral vectors bearing resistance to different antibiotics, such that heterozygosity of 2 alleles could be modeled. In concordance with an AR inheritance, the additional presence of a WT allele in cells carrying R223Q rescued the function to WT-levels of ISG induction (Figure 3D).

Finally, we compared the R223Q STAT2 allele reported herein to the R148Q STAT2 variant previously reported by our group (Gruber et al., 2020). Although the clinical disease arising from both STAT2 variants involved similar pulmonary and neurologic sequelae, the two alleles appear to manifest with variable clinical severity. We hypothesized that a direct genotype-phenotype relationship exists, whereby STAT2 R223Q (with survival in 1/3, and delayed lethality in 2/3 siblings) is milder than the previously-reported R148Q variant (with lethality in infancy for 1/1 confirmed cases, and similar early-onset deaths in 2 siblings without confirmed genotyping). However, both variants induced a similar magnitude of enhanced ISG expression after prolonged IFN-I stimulation (Figure 3E). We therefore tested whether these STAT2 variants differ not by the peak of their IFN-I response, but by the recovery time to fully terminate signaling. To examine these kinetics, cells were pulsed with IFN-I (12 hours), then sampled for qPCR analysis in a serial fashion until ISG transcripts return to unstimulated levels (Figure 3F). As compared to WT fibroblasts, which return to baseline ISG expression at around 72 hours, cells harboring either STAT2 variant continued to express ISG transcripts for several days. However, by 5 days, R223Q cells returned to baseline, while R148Q cells continue to express ISG transcripts (Figure 3F). These data suggest a model in which severity of autoinflammation across STAT2 alleles is governed by the time to termination of signaling, for which mutants that confer slow resolution of IFN-I signaling lead to greater accumulation of inflammatory-mediated damage.

### STAT2 R223Q binds USP18 but fails to recruit it to the IFNAR complex

**Figure 4:**
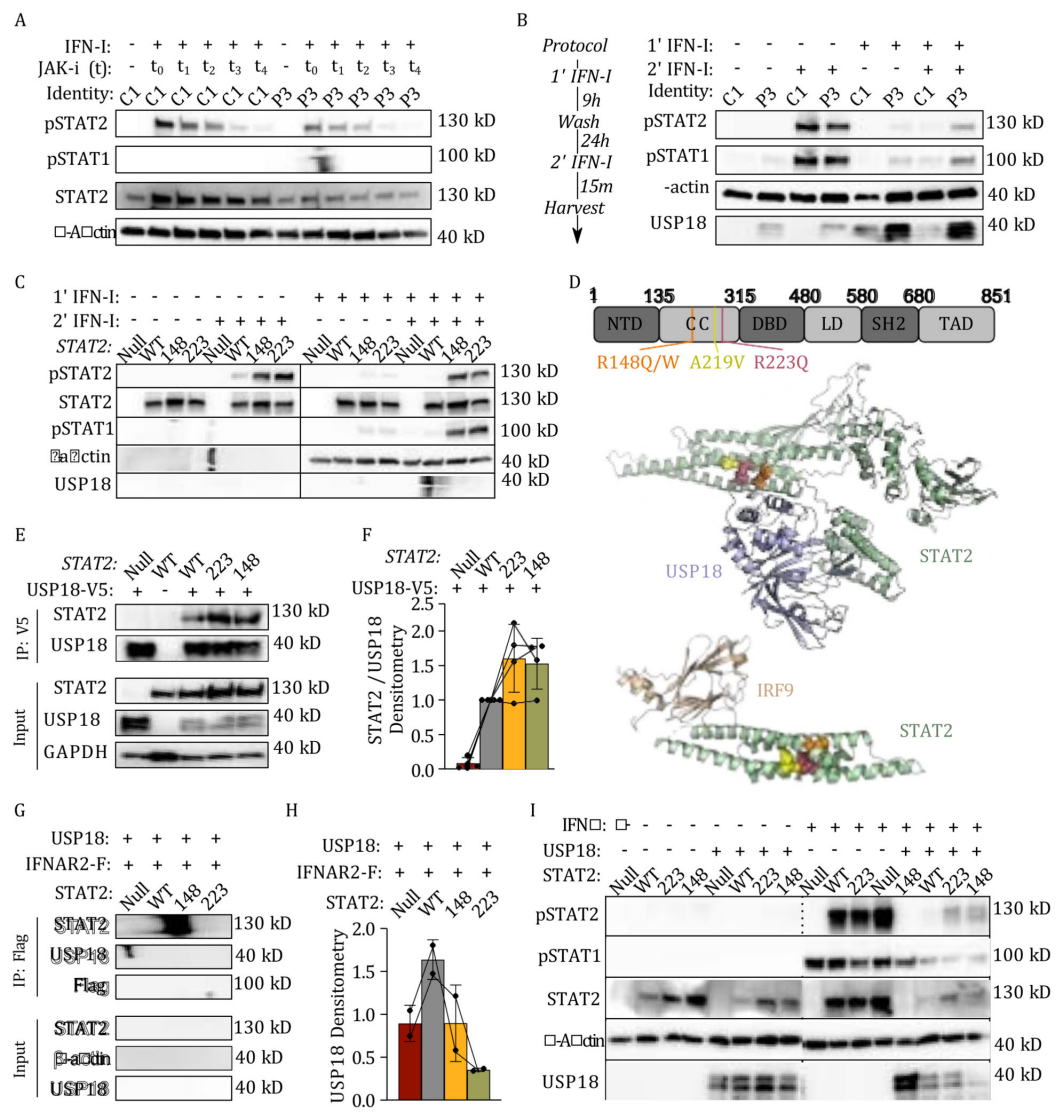
STAT2 R223Q fails to recruit USP18 to IFNAR2 for inhibition. (A) STAT Dephosphorylation rates in fibroblasts briefly stimulated with IFN-I (100 IU/mL) then treated with ruxolitinib and sampled serially over time (times points t_0_ – t_4_ correspond to 0, 30, 60, 120 and 180 minutes respectively). (B) Assessment of signaling refractoriness in dermal fibroblasts. Cells were initially treated with 100 IU/mL IFN-I or vehicle (“primed”) for 9-hours, washed then allowed to rest for 24 hours, at which time a secondary stimulus (“re-stim”) 100 IU/mL IFN-I or vehicle was given for 15 minutes. (C) Assessment of signaling refractoriness in STAT2-reconstituted U6A cells, as above. (D) Co-localization of activating STAT2 variants in the linear structure of STAT2 (top), the interaction domain between STAT2 and USP18 by *in silico* prediction (middle), and the association with the STAT2 CCD and IRF9 (bottom). (E) Analysis of USP18-STAT2 binding by immunoprecipitation of USP18-V5 in reconstituted U6A cells. (F) Quantification of the above, expressed as the quotient of the STAT2 signal in the IP over the USP18 signal in the IP. Points represent cumulative data from four independent experiments. Bars indicate mean ± SEM. (G) Efficiency of USP18 recruitment by immunoprecipitation of IFNAR2-Flag and immunoblotting for the presence of USP18 in in reconstituted U6A cells. (H) Quantification of above, expressed as the quotient of USP18 signal in the IP over the USP18 signal in the input. Points represent data from two independent experiments. Bars indicate mean ± SEM. (I) Capacity for ectopic USP18 to inhibit STAT phosphorylation by immunoblotting in IFN-I stimulated (100 IU/ml, 15 minutes) U6A cells reconstituted with the indicated *STAT2* alleles. Representative results of at least three independent experiments are shown for each panel, unless otherwise indicated.

We next reasoned that, in order to increase ISG expression at late timepoints, an activating variant would need to either inhibit STAT dephosphorylation or perturb the negative regulatory capability of STAT2. To assess dephosphorylation of STAT2, fibroblasts were stimulated briefly with IFN-I, washed, then treated with the JAK-inhibitor ruxolitinib so as to isolate the rate of dephosphorylation from further JAK-mediated phosphorylation. This study demonstrated normal rates of dephosphorylation in cells containing the mutant STAT2 allele (Figure 4A).

Next, negative regulation was assessed by comparing the capacity for restimulation (i.e. secondary stimulus) after an initial IFN-I exposure (primary stimulus). At such late timepoints, there is induction of USP18, which acts to inhibit further signaling after it is shuttled to the IFN-I receptor by STAT2. In healthy donor cells, this inhibition is readily demonstrated as ‘primed’ cells are unable to adequately respond to a secondary stimulus (Figure 4B). In contrast, patient fibroblasts maintained the ability to mediate a second response despite higher levels of USP18, suggesting a defect in USP18- mediated inhibition (Figure 4B). These data were recapitulated in STAT2-transduced U6A cells, with the R223Q STAT2 variant behaving similarly to the previously-reported R148Q mutant (Figure 4C). Likewise, in EBV-immortalized B-cells, which constitutively express USP18 (Supplementary Figure 4A), patient cells hyper-respond to IFN-I stimulation (Supplementary Figure 4B).

Given the shared functional consequences of the R223Q and R148Q alleles, we next analyzed the structural relationship of all IFNopathy-associated STAT2 variants reported to date (Gruber et al., 2020, Duncan et al., 2019, Zhu et al., 2023). Namely, the R223Q, R148Q, R148W, and A219V variants all impair function with USP18. In accordance, all variants co-localize within STAT2 to the coiled-coiled domain (CCD) domain (Figure 4D, top). Together with the DNA-binding domain (DBD), the CCD is essential for USP18-mediated signaling inhibition (Arimoto et al., 2017). *In silico* projection of the predicted USP18 and STAT2 interaction further reveals co-localization to the STAT2-CCD/USP18 interface (Figure 4D, middle). By contrast, the STAT2 mutants locate distally from the interface with IRF9 (Figure 4D, bottom). These data substantiate the preserved ISGF3 function of these STAT2 alleles and support the isolated loss of USP18-mediated negative regulation.

Previous reports have demonstrated that these STAT2 variants can lose inhibitory function by either impaired STAT2-USP18 binding (Duncan et al., 2019) or inadequate receptor localization of USP18 with retained STAT2-USP18 binding (Gruber et al., 2020). For R223Q STAT2, binding to USP18 was similar to WT STAT2 when studied by co-immunoprecipitation (Figure 4E and F). However, when localization of USP18 to IFNAR2 was assessed, R223Q STAT2 led to reduced USP18 receptor localization vis a vis WT, with levels comparable to the STAT2-null condition (Figure 4G and H). To confirm that the STAT2-USP18 axis was sufficient to account for these functional differences in signaling, we then co-transfected USP18 with WT or mutant STAT2, so as to directly assess the ability of STAT2 to facilitate USP18 inhibitory function. In line with the re-stimulation experiments, USP18 was unable to effectively inhibit an IFN-I stimulus in the presence of R223Q STAT2 (Figure 4I). Together, these findings indicate that R223Q STAT2 is unable to effectively traffic USP18 to its site of action at the receptor, thereby disrupting late termination of IFN-I signaling.

### Excessive type I IFN inflammation antagonizes monocyte chemotaxis, phenocopying CCR2-/-

**Figure 5:**
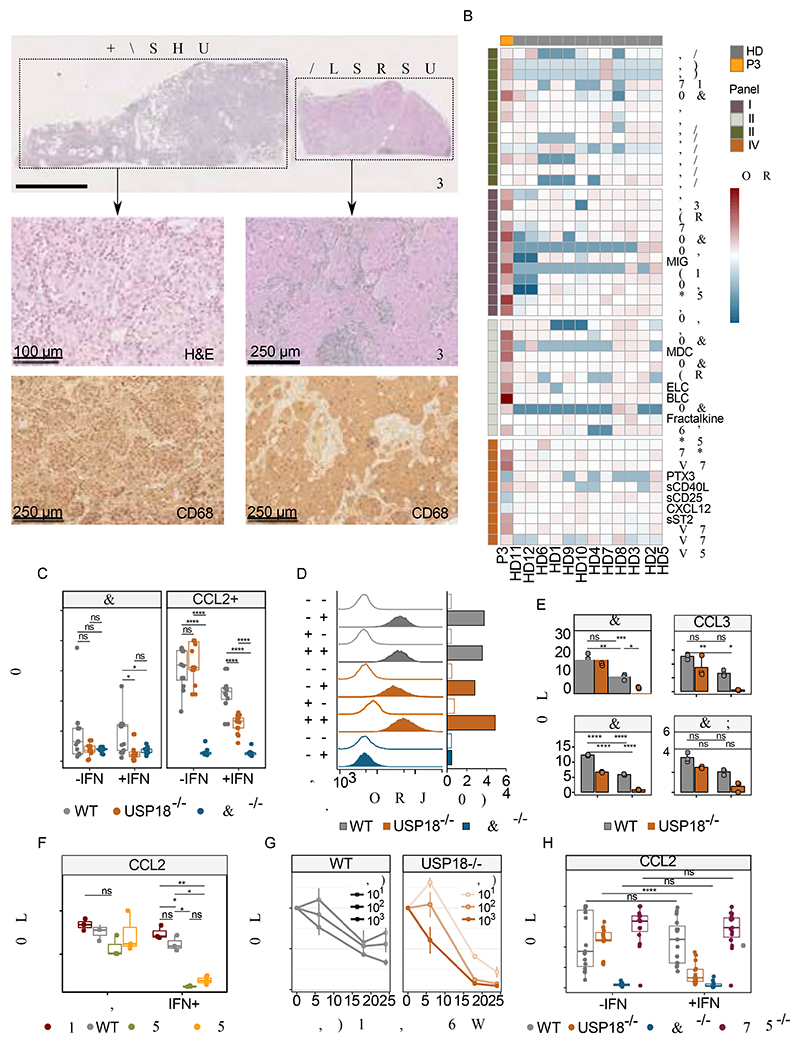
Interminable IFN-I signaling compromises monocyte chemotaxis (A) Lung biopsy from P1 stained with H&E, PAS or anti-CD68 antibodies to demonstrate the hypercellular areas (left) adjacent to the lipoproteinaceous areas (right). The corresponding distance represented by each scale bar is indicated on the image. (B) Circulating cytokines from the BAL fluid P3 (n=1) compared to healthy donors (HD1-12, n=12) by Luminex assay. Data represents a single experiment. (C) Transmembrane migration toward CCL-2 (20 ng/ml) in THP-1 cells (WT, USP18-/-, or CCR2-/-) previously treated with IFN-I (100 IU/mL, 8-hour stimulation, 36-hour rest). Migration expressed as a scaled value relative to maximum within each sample batch. Cumulative data from five independent experiments are shown (n=2-3 biological replicates per experiment, total n=13 per sample). (D) Surface expression of CCR2 by flow-cytometry using PE-conjugated CCR2 antibodies (α-CCR2) or an isotype control. Prior to labeling, THP-1 cells were stimulated with IFN-I (100 IU/mL, 8-hour stimulation, 36-hour rest). Results represent data from two independent experiments (E) Transwell migration in WT or USP18-/- THP-1 cells in response to CCL-2, CCL-3, CCL-5, or CXCL-12. Migration expressed relative to unstimulated WT condition in the absence of chemokine. Bars indicate mean ± SEM. Data are representative of three independent experiments performed with biological replicates (n=3). (F) CCL-2 directed chemotaxis after 48-hours of IFN-I treatment in STAT2-/- THP-1 cells transduced with either: empty vector (null), WT STAT2, R148Q STAT2 or R223Q STAT2. Data are representative of three independent experiments performed with biological replicates (n=3). (G) THP-1 migration in response to increasing duration and doses of continuous IFN-I stimulation. Migration expressed relative to unstimulated condition. Data are representative of two independent experiments performed with biological replicates (n=3). (H) Migration of TREX1-/- THP-1 cells in response to CCL-2 as compared to WT, USP18-/-, or CCR2-/- background. Migration expressed as a scaled value relative to maximum within each sample batch. Cumulative data from five independent experiments are shown (n=3 biological replicates per experiment, total n=15). Statistical testing was performed with one-way ANOVA followed by the Holm-Sidak’s test for multiple comparisons. P-values are abbreviated *P < 0.05, **P < 0.01, ***P < 0.001, or ns = not significant *P > 0.05*.

Understanding that clinical disease in three siblings arose from over-exuberant host IFN-I responses to infectious stimuli, rather than a true infectious susceptibility, we next sought to elucidate the pathogenesis of the observed pulmonary disease. The cytopathological analysis of a lung biopsy performed for P1 at 17 months showed features consistent with PAP, a unique disorder marked by accumulation of lipo-proteinaceous pulmonary surfactants within alveoli (Figure 5A, Supplementary Figure 5A). However, unlike in typical PAP, these findings were distributed heterogeneously within the lung parenchyma: some regions demonstrated eosinophilic PAS-positive air-spaces characteristic of PAP (lipoproteinaceous areas), while adjacent zones demonstrated an atypical infiltration of numerous macrophages (hypercellular areas) (Figure 5A, Supplementary Figure 5A). The macrophages in these hypercellular areas lacked the enlarged, foamy appearance typically observed in PAP. Interestingly, a similar PAP phenotype was observed in the initial reporting of the R148Q STAT2 variant (Gruber et al., 2020), however the mechanistic link to excessive IFN-I activity remains unknown. To date, all described etiologies of immune-mediated PAP compromise monocyte/macrophage clearance of alveolar surfactant. These include hereditary forms due to variants affecting GM-CSF signaling (*CSF2RA, CSF2RB, STAT5B, OAS1*) (Krone, 2019, Tanaka et al., 2011, Suzuki et al., 2008, Magg et al., 2021), myeloid lineage development (*GATA2, ADA1, ADA2, IRF8*, and *SLC7A*) (Marciano et al., 2021, Grunebaum et al., 2012, Zhang and Cao, 2017, Rosain et al., 2022), and monocyte chemotaxis (*CCR2*)(Neehus et al., 2023). Likewise, acquired forms exist, due to high-titer neutralizing GM-CSF auto-antibodies (McCarthy et al., 2022).

We first profiled the cytokine signature of BAL fluid (Figure 5B) and peripheral blood of P3 (Supplementary Figure 5B). This analysis demonstrated a broadly inflamed cytokine milieu, with intact circulation of chemokines essential to monocyte trafficking (e.g. CCL2, CCL7, CCL8, CXCL12). Additionally, high levels of IFNα protein were detected in BAL fluid by single molecule array (SIMOA) digital immunoassay (1166 fg/ml) (Rodero et al., 2017). We next assessed for autoimmunity against GM-CSF in P3, given the presence of other autoantibodies in the clinical evaluation (see Case Report) and in IFNopathies more generally (Gagne et al., 2023). No evidence for neutralizing auto-antibodies to GM-CSF was detected, as compared to positive control samples from patients with known GM-CSF autoimmunity (Supplementary Figure 5C). We next examined the GM-CSF/STAT5 signaling axis for antagonism by excessive IFN-I signaling. By CRISPR knockout of *USP18* in a monocytic cell line, THP-1, we experimentally modeled the most severe form of activating *STAT2* variants (Supplementary Figure 5D-E). Whether or not cells were primed with IFN-I, *USP18-/-* cells demonstrated intact signaling responses to GM-CSF stimulation (Supplementary Figure 5F), suggesting against a GM-CSF-signaling defect.

Recently, it was discovered that PAP can also arise from genetic deficiency of CCR2 (Neehus et al., 2023, Cakar et al., 2025, Herkner et al., 2025), the receptor for CCL2 and other ligands, which together constitute an essential axis for monocyte chemotaxis to the lung. First, we confirmed that no auto-antibodies against CCL-2 or other CCR2-mediated cytokines were detected in serum from P3 (data not shown). To assess chemotactic function in response to CCL-2, migration through a permeable membrane was measured in WT, *USP18*-/-, or *CCR2*-/- THP-1 cells. To recapitulate *in vivo* conditions, cells were initially primed with IFN-I, then rested in the absence of IFN-I to allow for USP18/STAT2-mediated signal resolution. As expected, *USP18*-/- cells migrated similarly to their WT counterpart in the absence of IFN-I (Figure 5C). However, priming with IFN-I led to a drastically reduced migratory capacity of USP18-/- cells, approaching the unresponsiveness observed in *CCR2*-/- cells.

To probe the mechanisms underlying this broad chemotaxis defect, we next tested the hypothesis that reduced chemokine receptor expression drives the impairment in chemotaxis. In both the IFN-naïve and IFN-primed state, WT and USP18-/- cells exhibited similar surface expression of CCR2 by flow cytometry (Figure 5D), suggesting against a mechanism mediated by differential receptor expression. Likewise, signaling of pathways downstream of the chemokine receptor (pERK2 and pAKT) were similar between cell types (Supplementary Figure 5G).

Next, we assessed whether this chemotaxis defect arising from prolonged IFN signaling extends to chemokines other than CCL-2. Using chemokines that utilize a broad array of cognate receptors (CCR1, CCR3, CCR5, CXCR4) (Hughes and Nibbs, 2018), we observed a similar IFN-mediated antagonism of chemotaxis toward CCL-3, CCL-5, and CXCL-12 (Figure 5E). These findings suggest that prolonged IFN-I stimulation antagonizes chemotactic mediators or migratory pathways that are shared across diverse chemokines.

We then validated that the migratory phenotype observed in USP18-/- THP-1 cells can be recapitulated in monocytes harboring the STAT2 R223Q variant. We knocked out *STAT2* in THP-1 cells and reconstituted their genotype via lentiviral transduction with either an empty vector, WT STAT2, R148Q STAT2 or R223Q STAT2. As compared to cells expressing WT STAT2, those carrying either R223Q or R148Q STAT2 demonstrated enhanced ISG expression (Supplementary Figure 5H) but reduced migration (Figure 5F) in response to prolonged IFN-I exposure.

Finally, we aimed to understand why PAP appears unique to genetic defects of IFN-I signaling regulation. By contrast, neither the other genetic IFNopathies that arise from overproduction of IFN-I nor therapeutic IFN-I administration for immunologic or oncologic indications have been associated with PAP. These observations suggest an essential role for negative regulation of the IFN-I response in the homeostasis of monocyte migration. Of note, slightly reduced chemotaxis with IFN-I-priming could be detected in WT cells, albeit to a smaller magnitude than in USP18-/- cells (Figure 5D). In accordance, minimal residual ISG expression was detected in WT cells as compared to USP18-/- cells with prolonged stimulation (Supplementary Figure 5E). We therefore hypothesized that loss of IFN-I negative regulation leads to an accumulation of ISG activity that antagonizes monocyte migration beyond a threshold of clinical significance. Consistent with this notion, a dose- and time-dependent relationship between IFN-I stimulation and migratory inhibition could be readily observed (Figure 5G). Notably, USP18-/- cells exhibited a disproportionate reduction in migration with increasing IFN-I concentrations and prolonged stimulation (Figure 5G). We then investigated migration in THP-1 cells deficient for *TREX1*, a common genetic etiology of IFNopathy, which results in the overproduction of IFN-I without disruption of the downstream regulatory response. Unlike USP18-/- cells, TREX1-/- THP-1 cells exhibited only minimal antagonism with exogenous IFN-I stimulation, in accordance with their intact USP18-mediated regulation (Figure 5H). This observation suggests that negative regulation buffers against ISG-driven migratory defects in the context of *TREX1* deficiency and other related IFNopathies, potentially explaining the absence of PAP.

In sum, these experiments demonstrate that, for patients with USP18/STAT2-driven IFNopathy, prolonged IFN-I signaling antagonizes monocyte chemotaxis toward chemokines implicated in alveolar homeostasis, among which the CCL2/CCR2-axis deficiency is known to drive PAP (Neehus et al., 2023).

### Pharmacologic JAK and IFNAR1 inhibition improve clinical disease

**Figure 6:**
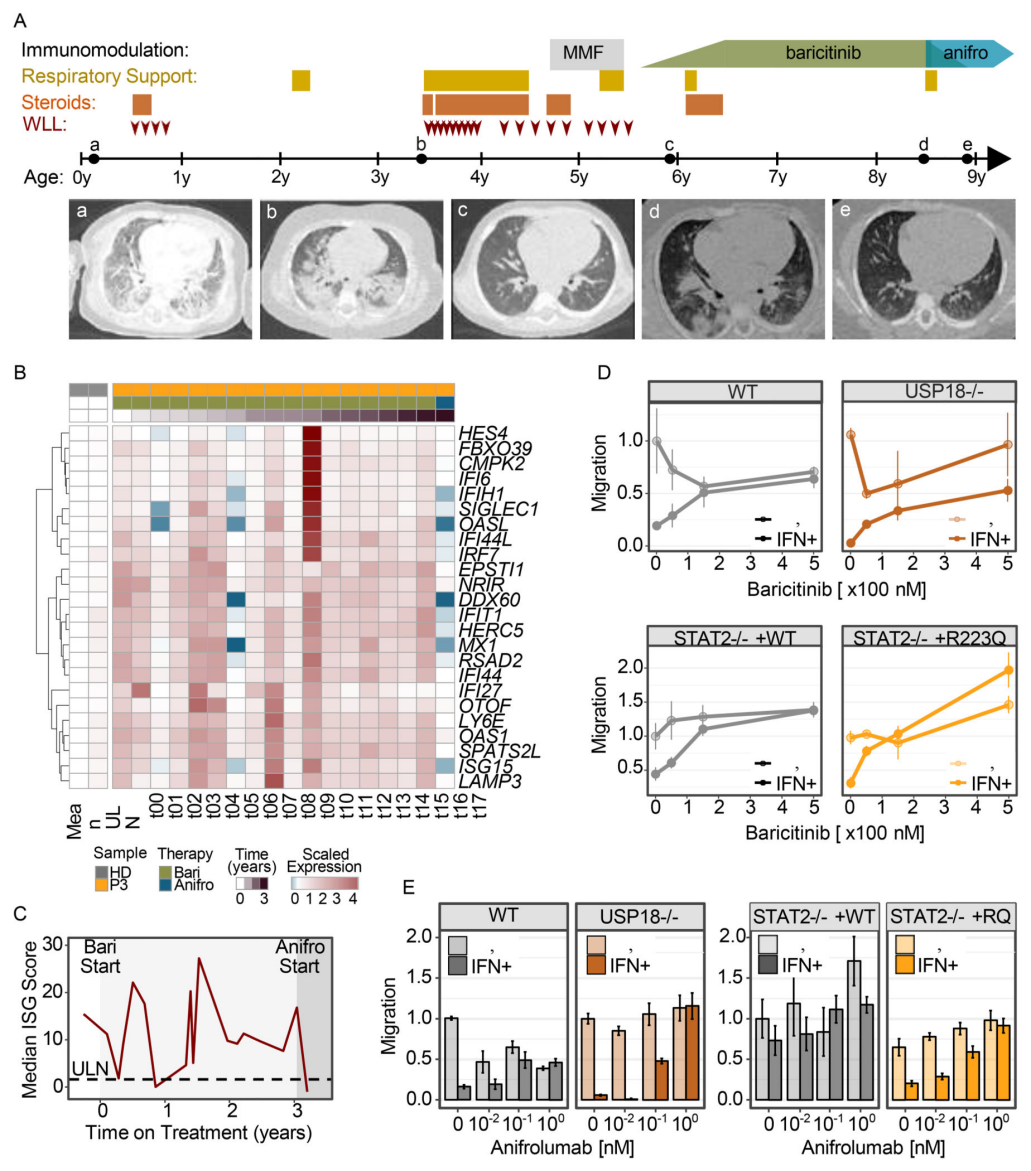
JAK- and IFNAR1- inhibition improve pulmonary and neurologic disease in P3 (A) Schematic representation of approximate timeline of clinical therapies before and after baricitinib and anifrolumab treatment. Lower panel demonstrates radiologic improvement in pulmonary disease by serial CT scans. (B) ISG levels by Nanostring in peripheral blood from P3 over the time of treatment (t0 = initiation of baricitinib) as compared to healthy donors (expressed as mean and upper limit). Data represents a single experiment. (C) Median ISG score over time. Data derived from the Nanostring expression profile (also shown in Figure 5B). (D) Effect of baricitinib treatment (48 hours at indicated dosing) on IFN-mediated antagonism of chemotaxis in USP18-/- (top) and STAT2-reconstituted (bottom) THP-1 cells, as compared to their WT counterparts. Data are representative of at least two independent experiments performed with biological replicates (n=3). (E) Anifrolumab treatment (24 hours) in IFN-treated (100 IU/mL) USP18-/- (left) and STAT2-reconstituted (right) THP-1 cells, as compared to their WT counterparts. R223Q abbreviated RQ. Data are representative of at least two independent experiments performed with biological replicates (n=3).

Prior to genetic diagnosis, P3 underwent several unsuccessful trials with conventional immunosuppressive therapies (Figure 6A). This included a 1-year course of mycophenolate mofetil (MMF), during which an increasing frequency of WLL treatment was required, often in excess of monthly intervals. Corticosteroids were also administered intermittently for respiratory decompensations and acute meningoencephalitis – affording only minimal stabilization in each case (Figure 6A). Guided by the aforementioned molecular studies, the JAK inhibitor baricitinib was initiated in P3 at age 5 years. Dosing was started at 2mg three times daily. IVIG was administered in tandem for varicella zoster virus (VZV) prophylaxis, given his unimmunized status against VZV, as well as the well-documented increased risk of VZV disease associated with JAK inhibition.

At the time of initiation of baricitinib, P3 required supplemental oxygen and monthly therapeutic WLL. By 3 months of treatment, there was marked improvement in his respiratory status and WLL treatments were able to be discontinued (Figure 6A). An interval chest CT scan at 4 months post-initiation demonstrated improvement, as evidenced by the regression of alveolar consolidation and ground glass opacities compared to prior studies (Figure 6A). Thereafter, the frequency and intensity of respiratory decompensations with infectious episodes drastically improved. Likewise, a marked neurologic recovery was observed, with only minimal disease relapse occurring after baricitinib dosing was briefly held. Namely, the patient experienced a neuroinflammatory flare, thought to be viral-induced, although without an identifiable viral agent. Corticosteroids were re-initiated but later tapered off with a dose change in baricitinib to 4 mg twice daily.

Serial analysis of the ISG response was performed throughout the treatment course. In peripheral blood mRNA, ISG expression decreased over the initial few months of baricitinib therapy, parallel to clinical improvement (Figure 6B-C). However, with longer duration of monitoring, ISG levels fluctuated markedly, at times returning to pre-treatment values. This discordance suggests that clinical improvement can occur without complete biochemical rescue (ISG expression), as often observed across the IFNopathies (Sanchez et al., 2018, Fremond et al., 2021, Li et al., 2022, Vanderver et al., 2020). Alternatively, these data may indicate that residual disease activity persists, which could potentiate future clinical relapse.

Unfortunately, despite a prolonged period of clinical stability on baricitinib, the patient experienced a relapse in pulmonary disease after approximately 1.5 years of therapy. He acutely redeveloped a requirement for supplemental oxygen, which correlated with a rise in peripheral blood ISG levels (Figure 6B-C) and recrudescence of alveolar consolidations and multifocal ground glass opacities by cross-sectional imaging. No inciting infectious agent was identified.

Anifrolumab, a monoclonal antibody targeting IFNAR1, was subsequently initiated at a dosing of 300mg every 4 weeks. In tandem, baricitinib was progressively tapered off over a 6-month transition period. The patient demonstrated improvement in his respiratory status both clinically and radiologically (Figure 6A). At the last evaluation, after 7 months of anifrolumab therapy, the patient remains off respiratory support, WLL and corticosteroid therapy, and continues to demonstrate progressive neurologic and pulmonary improvement. In accordance, a normalization of ISG scores from peripheral blood has been observed (Figure 6B-C).

To demonstrate that the clinical benefit from anifrolumab and baricitinib tracks with rescue of the monocyte chemotaxis defect, we tested the efficacy of these therapeutics in the THP-1 migration system. In both the USP18-/- and STAT2-reconstituted model, baricitinib treatment led to a dose-dependent recovery in monocyte migration from IFN-mediated antagonism (Figure 6D). Likewise, anifrolumab demonstrated a similarly effective reversal of the pathogenic phenotype (Figure 6E). These data, taken together with the clinical observations above, provide further evidence for the role of unregulated IFN-I activity in the development of PAP.

## Discussion

This report adds a novel genetic mutation to a recently-discovered disease entity whereby biallelic *STAT2* variants underlie a type I IFNopathy, termed here Type-I Interferonopathy by Mutation of *STAT2* (TIMS2). In all four deleterious variants reported to date, impaired trafficking of USP18 by mutant STAT2 prevents IFN-I signal termination (Duncan et al., 2019, Gruber et al., 2020, Zhu et al., 2023). The resulting inflammatory disease clinically and biochemically phenocopies the IFNopathy of ISG15- or USP18- deficiency (Martin-Fernandez et al., 2022, Meuwissen et al., 2016, Zhang et al., 2015). Across these disorders, a common neurologic, dermatologic and pulmonary phenotype is shared, albeit with variable expression.

This case demonstrates that hyperinflammatory disease can masquerade as apparent susceptibility to infection. However, unlike in STAT2 LoF variants that lead to a *bona fide* immunodeficiency in control of viral replication (e.g. viral pneumonia), the TIMS2 variants confer normal antiviral activity. Rather, infection-associated disease (e.g. PAP) likely arises from an inability to curb the inflammatory-mediated damage from transient infectious stimuli. Additionally, this unrestrained inflammation may engender a secondary immunodeficiency. For example, we have previously demonstrated that excessive IFN-I can disrupt the IFN-II/IL-12 axis essential for mycobacterial immunity (Martin-Fernandez et al., 2022) and compromise tissue repair (Sazeides et al., 2026). Nonspecific factors of severe illness that increase infectious risk (e.g. organ failure, mechanical ventilation, nosocomial exposures, and corticosteroid use) could also contribute. In considering the sum of these processes together with the overexuberant inflammatory responses and documented signs of autoimmunity, it may be most apt to consider TIMS2 as a disease of immune dysregulation.

For TIMS2 patients, inflammatory disease manifested primarily as PAP, a rare lung disease of impaired surfactant clearance. In classical forms of immune-mediated PAP, genetic or acquired lesions in the GM-CSF pathway impair macrophage function, leading to the accumulation of large foamy macrophages and amorphic PAS+ material in the alveoli. By contrast, the STAT2/USP18-axis defects described herein, demonstrate intact intrinsic GM-CSF signaling and an absence of anti-GM-CSF autoimmunity. Further, affected lung tissue in TIMS2 exhibited an atypical histological pattern, with marked heterogeneity of classical PAS+ zones intermixed with hypercellular regions of macrophages lacking morphologic features characteristic of PAP. This observation could suggest a disorganization of monocyte/macrophage trafficking in the lung *in vivo*. Accordingly, we demonstrate that prolonged IFN-I signaling directly antagonizes *in vitro* chemotaxis toward essential lung chemokines, including CCL-2. This mechanism partially phenocopies the recently-discovered human deficiency of the CCL-2 receptor (CCR2), which manifests with PAP, polycystic lung disease and infection (Neehus et al., 2023). In this alternative etiology of PAP, GM-CSF-driven macrophage function is retained, but monocyte entry into the alveolar space is precluded. Consequently, the CCR2-deficient lung environment uniformly lacks foamy macrophages, unlike in the heterogenous tissue of TIMS2. Conceivably, this difference may arise from the broad antagonism across various chemokines in TIMS2, as compared to the narrow CCL-2 defect in CCR2 deficiency. Additionally, macrophage mislocalization or other secondary effects may result in GM-CSF-independent phagocytic defects in TIMS2, ultimately leading to foamy macrophages.

Importantly, a clear dose- and time-dependent relationship exists between IFN-I activity and defective chemotaxis. Unlike with defective STAT2/USP18-mediated regulation, systems with intact IFN-I negative feedback manifest only mild antagonism of chemotaxis by IFN-I, presumably below a theoretical threshold for clinically-significant consequences. This observation likely explains the lack of PAP in routine viral infections in healthy individuals, during exogenous IFN-α therapy for oncologic indications, and in ‘traditional’ IFNopathies that arise from over-production of IFN-I. Interestingly, GoF variants in *OAS1*, the intracellular double strand RNA sensor, have been associated with PAP and IFN-mediated hyperinflammation. However, the clinical phenotype does not resemble an IFNopathy, and the molecular pathogenesis involves impaired GM-CSF signaling directly related to translational arrest from OAS1 activation of RNAse L (Magg et al., 2021). In any case, future studies should explore IFN-I in the pathogenesis of other PAP etiologies that remain poorly understood. These include complex infections, systemic juvenile idiopathic arthritis, and Down syndrome—all of which are known to dysregulate IFN-I activity.

There exist three outstanding questions regarding the molecular and clinical pathogenesis of TIMS2. First, it remains unclear how certain STAT2 variants (R148Q and R223Q) retain their binding with USP18 yet fail to traffic it appropriately to IFNAR2. While other pathogenic STAT2 variants (R148W and A219V) of the CCD demonstrate a direct loss in USP18 binding (Duncan et al., 2019, Zhu et al., 2023), experimental deletion of the entire CCD does not appreciably affect STAT2-USP18 coimmunoprecipitation (Arimoto et al., 2017). Although some of these discrepancies may arise from technical differences, collectively these findings suggest that the STAT2–USP18 interaction likely involves more complex mechanisms than simple direct binding. Possibly, an additional adapter protein in the STAT2-USP18 complex is required for negative regulation. Forward and reverse genetic studies with TIMS2 patients and in vitro models will be invaluable to better understanding this essential regulatory hub.

Second, the mechanisms by which excessive IFN-I disrupts chemotaxis are incompletely defined. We have demonstrated thus far that neither chemokine receptor levels nor AKT/ERK signaling can explain this phenomenon. However, chemotaxis relies on a remarkably broad network of signaling cascades, many of which have yet to be investigated in this context. We hypothesize that a signaling intermediate shared across chemokine pathways is directly or indirectly regulated by ISG activity. Unraveling these molecular determinants will be key in future investigations.

Third, as cases of TIMS2 start to accumulate, heterogeneity in clinical severity is increasingly observed. The initial reports in R148Q and R148W STAT2-carrying individuals documented lethality around infancy. In contrast, a 23-year-old patient recently reported to harbor an A219V mutation suffered severe albeit non-fatal disease. The patients reported herein had variable outcomes, ranging from death in early infancy to survival to 5 years of age (at which time baricitinib was started) and beyond—currently 9 years-old at the time of reporting. This range likely reflects both intrinsic and extrinsic factors. As compared to the invariably lethal mutation of R148, the R223Q allele exhibited earlier resolution of IFN-I signaling. Yet, outcomes differed even among siblings carrying the R223Q allele. Notably, those with very early and/or frequent infectious exposures succumbed to death, suggesting a key role for exogenous immune stimulation. Likewise, early lethality in R148W STAT2 was associated with *in utero* exposure to Influenza (Duncan et al., 2019). These extrinsic factors, which drive periods of IFN-I activation and delayed signal resolution, may also explain the fluctuating ISG levels observed in P3 after treatment (Figure 6C). Taken together, clinical severity can therefore be conceptually modelled as a function of cumulative inflammation from both the frequency of immune stimulation and the kinetics of its resolution.

Finally, the intricate mechanisms of TIMS2 pose a unique challenge as to whether to classify these mutations as GoF (for the overall effect on IFN signaling) or LoF (relative to its negative regulatory capacity). While this traditional dichotomy is practical for proteins with a single function, it becomes difficult when considering proteins like STAT2 with dual, but opposing, functions (i.e. signal transduction and negative regulation). For the above reasons, we advocate for an unambiguous approach that avoids the LoF/GoF designation in favor of language that emphasizes the net inflammatory disease phenotype: TIMS2. Beyond *STAT2*, this approach is likely to be useful as the booming discovery of human mutations yields other single gene disorders with such nuanced molecular pathogenesis.

## Supplementary Material

Supplementary

Supplementary Figures

## Figures and Tables

**Table 1:** Clinical features in P1, P2 and P3.

	P1	P2	P3
**Demographics**			
Sex	Female	Female	Male
Birth Year	2011	2016	2017
Age at Report/Death	deceased, 19 months	deceased, 4 months	alive, 9 years old
**Pulmonary Alveolar Proteinosis**			
Disease Onset	4 months	3 months	4 months
BAL Gross Appearance	Milky	Milky	Milky
BAL Total Cell Count	150-500 / μL	1100 cells / μL	630-800 cells / μL
BAL Cell Differential	Neutrophil 10% Lymphocyte 14% Macrophage 76%	Neutrophil 12% Lymphocyte 3% Macrophage 85%	Neutrophil 17% Lymphocytes 3% Macrophages 80%
Biopsy Performed	Y	N	N
**Neurologic Disease**			
Clinical Presentation	Seizures, aseptic meningoencephalitis	none	Aseptic meningoencephalitis, developmental delay
CT Findings	NA	NA	Intracranial calcification
MRI Findings	Signal abnormalities of the brainstem and basal ganglia	NA	Hemispheric cortical atrophy
**Other Organ Systems**			
Autoantibody Serologies	+ANA, +smooth muscle, +thyroglobulin	+ANA: 1/1000	ANA: 1/1280 (peak), cANCA: 1/320 (peak)
Hepatic Involvement	Hepatomegaly, elevated AST/ALT	Hepatomegaly, elevated AST/ALT	none
Miscellaneous	Hypoglycemia	NA	Lymphadenopathy, polyphagia/obesity
**Treatment**			
Therapies	WLL, corticosteroids, cyclophosphamide	WLL, corticosteroids	WLL, corticosteroids, mycophenolate, baricitinib, anifrolumab

**Table 2:** Immunophenotyping of lymphocytes in P3. Absolute count and relative frequency (percent of lymphocytes) of lymphocyte subsets from P3 at age 5 years.

Lymphocyte Subset	P3 (reference range)
CD3+ (/μL)	4580 (1400-3700)
CD3+ (%)	64.4 (56-75)
αβ+ZCD3+ (%)	n.d.
γδ+ZCD3+ (%)	n.d.
CD3+CD4+ (/μL)	2574 (700-2200)
CD3+CD4+ (%)	36.2 (16-30)
CD45RO+ZCD4+ (%)	20.0 (14-47)
CD45RA+ZCD4+ (%)	80.0 (58-70)
CD31+CD45RA+ZCD4+ (%) *Recent thymic emigrant T CD4 cells*	64.3 (43-55)
CD3+CD8+ (/μL)	1899 (490-1300)
CD3+CD8+ (%)	26.7 (16-30)
CCR7+CD45RA+ZCD8+ (%) *Naïve T CD8 cells*	54.9 (52-68)
CCR7+CD45RA-ZCD8+ (%) *Central memory T CD8 cells*	0.4 (3-4)
CCR7-CD45RA-ZCD8+ (%) *Effector memory T CD8 cells*	24.2 (16-29)
CCR7-CD45RA+ZCD8+ (%) *TEMRA CD8 cells*	20.6 (11-20)
CD19+ (/μL)	2361 (390-1400)
CD19+ (%)	33.2 (6.1-25.2)
CD27+ (%)	8.7 (8.1-33.3)
IgD+CD27- (%)	87.6 (59.7-88.4)
IgD+CD27+ (%)	4.7 (3.1-18)
IgD-CD27+ (%)	4.0 (2.9-17.4)
CD24++CD38++ZCD19+ (%) *Transitional B cells*	0.1 (4.5-9.2)
CD24-CD38++ (%) *Plasmablasts*	0.4 (0.7-3.5)
CD21lowCD38dim (%) *Autoreactive B cells*	3.8 (0.9-3.5)
CD3-CD16+CD56+ (/μL)	171 (130-720)
CD3-CD16+CD56+ (%)	2.4 (4-17)

**Table 3:** *In silico* damage prediction scores for the c.668G>A STAT2 variant.

Prediction Tool	Version	Score Type	Value	Range	Interpretation
Alpha Missense	March 2025	Pathogenicity	0.112	0 - 1	Likely Benign
CADD	v.1.7, hg38	PHRED	22.5	1 - 99	Deleterious
MetaLR	dbNSFP v4.7c	Rank Score	0.40099	0 - 1	Tolerated
Mutation Assessor	dbNSFP v4.7c	Functional Impact Score	0.288	-5.17 - 6.49	Low Impact
MutationTaster	v.2	Probability	0.87	0 - 1	Polymorphism (probably harmless)
PolyPhen-2	v.2.2.3	Functional Impact Score	0.02	0 - 1	Benign
REVEL	v.1	Probability	0.055	0 - 1	NA
SIFT	v.6.2.1	Functional Impact Score	0.04	0 - 1	Deleterious

## Data Availability

Whole exome sequencing data was unavailable for deposition in public databases due to IRB protocol and patient privacy restrictions. All other raw data can be obtained from the corresponding author, D Bogunovic, upon reasonable request.

## Non-Standard Abbreviations

AKT: Protein Kinase B
ANA: Antinuclear Antibody
BAL: Bronchoalveolar Lavage
CADD: Combined Annotation Dependent Deletion
CCD: Coiled-coil domain
CCL2: C-C Motif Chemokine Ligand 2
CCR2: C-C Motif Chemokine Receptor 2
CSF: Cerebrospinal fluid
CT: Computed Tomography
DBD: DNA-binding domain
ERK2: Extracellular Signal-Regulated Kinase 2
GM-CSF: Granulocyte-Macrophage Colony-Stimulating Factor
GoF: Gain-of-Function
hTERT: Human Telomerase Reverse Transcriptase
ICU: Intensive Care Unit
IEI: Inborn Errors of Immunity
IFN-α: Interferon alpha
IFN-I: Type I interferon
IFNAR1: Interferon Alpha/Beta Receptor Subunit 1
IFNAR2: Interferon Alpha/Beta Receptor Subunit 2
IFNopathies: Type I Interferonopathies
IRF9: Interferon Regulatory Factor 9
ISG: Interferon stimulated gene
ISGF3: Interferon-Stimulated Gene Factor 3
JAK: Janus Kinase
LoF: Loss-of-Function
MAF: Minor Allele Frequency
MMF: Mycophenolate mofetil
MRI: Magnetic Resonance Imaging
PAP: Pulmonary alveolar proteinosis
PAS: Periodic Acid-Schiff
RSV: Respiratory Syncytial Virus
SAVI: STING-Associated Vasculopathy with onset in Infancy
STAT1: Signal Transducer and Activator of Transcription 1
STAT2: Signal Transducer and Activator of Transcription 2
TIMS2: Type-I Interferonopathy by Mutation of STAT2
TYK2: Tyrosine Kinase 2
USP18: Ubiquitin Specific Peptidase 18
WLL: Whole lung lavage.
