# Supplementary material for: Familial Pulmonary Alveolar Proteinosis and Type-I Interferonopathy by Mutation of STAT2 (TIMS2)
Source: J Exp Med. Author manuscript; Available in PMC 2026 Aug 11. (PMC7619351; doi:10.1084/jem.20251331)
Supplement: Supplementary file 1 [file EMS216003-supplement-Supplementary.pdf]

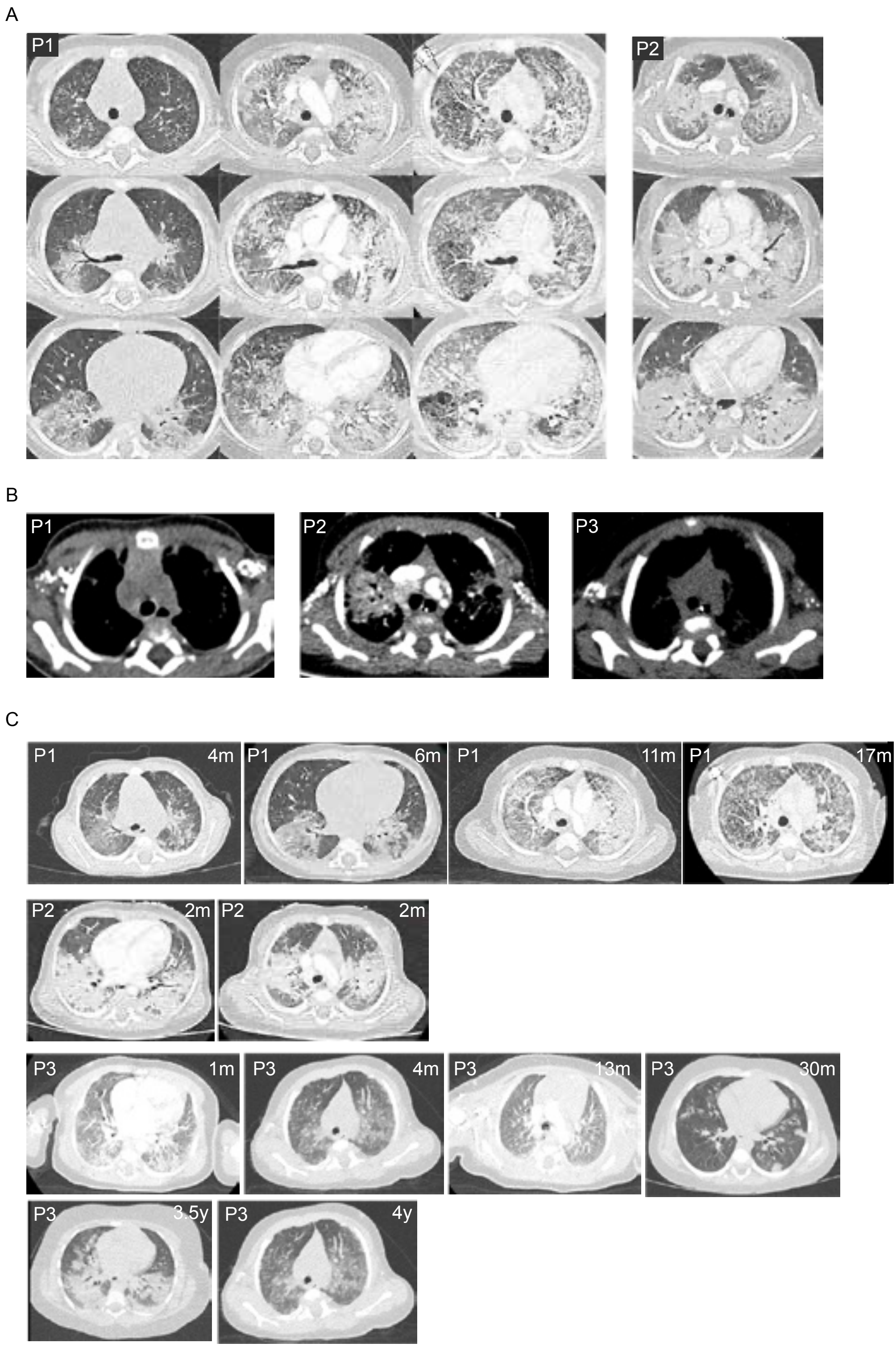
A
P1
P2
B
P1
P2
P3
C
P1 4m
P1 6m
P1 11m
P1 17m
P2 2m
P2 2m
P3 1m
P3 4m
P3 13m
P3 30m
P3 3.5y
P3 4y

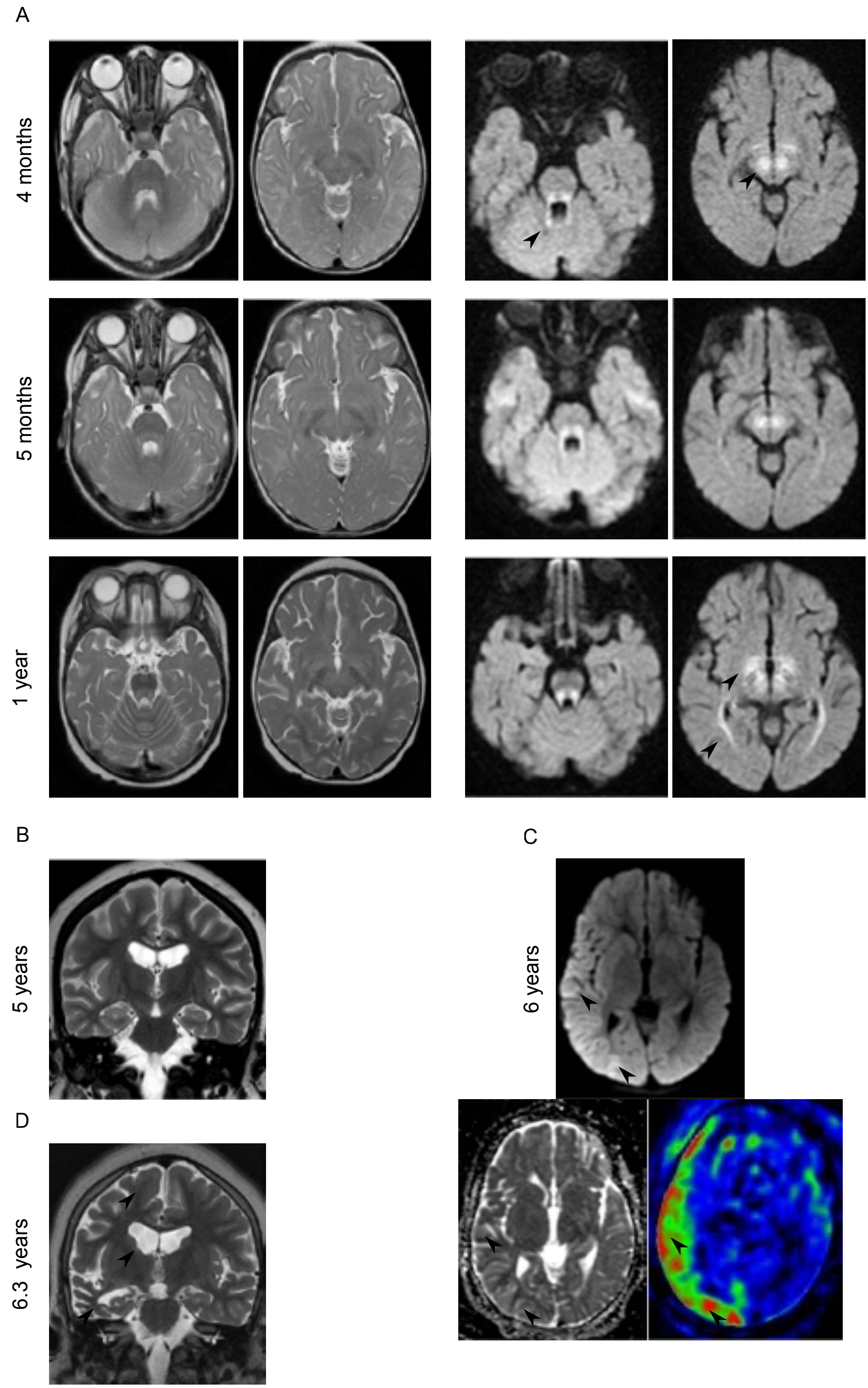
A
4 months
5 months
1 year
B
5 years
C
6 years
D
6.3 years

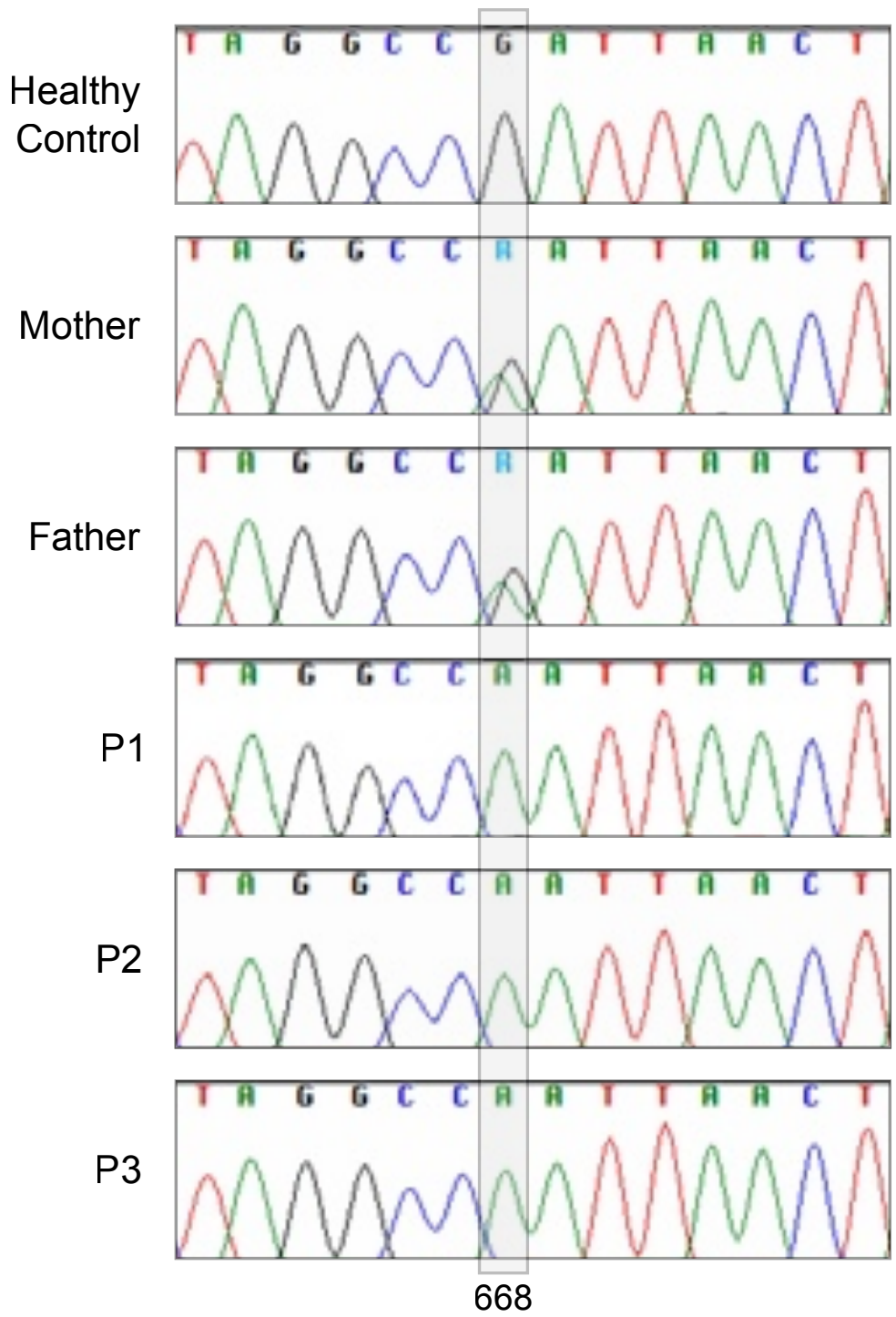
Healthy Control
Mother
Father
P1
P2
P3
668

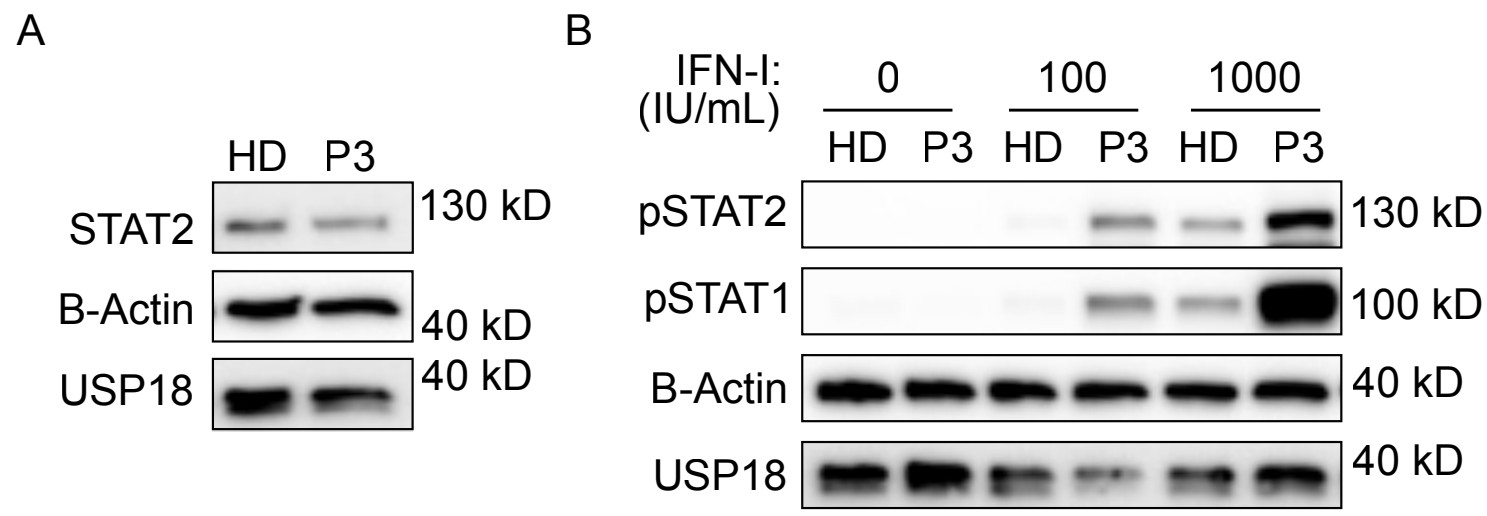
A
HD
P3
STAT2
130 kD
B-Actin
40 kD
USP18
40 kD
B
IFN-I:
(IU/mL)
0
100
1000
HD
P3
HD
P3
HD
P3
pSTAT2
130 kD
pSTAT1
100 kD
B-Actin
40 kD
USP18
40 kD

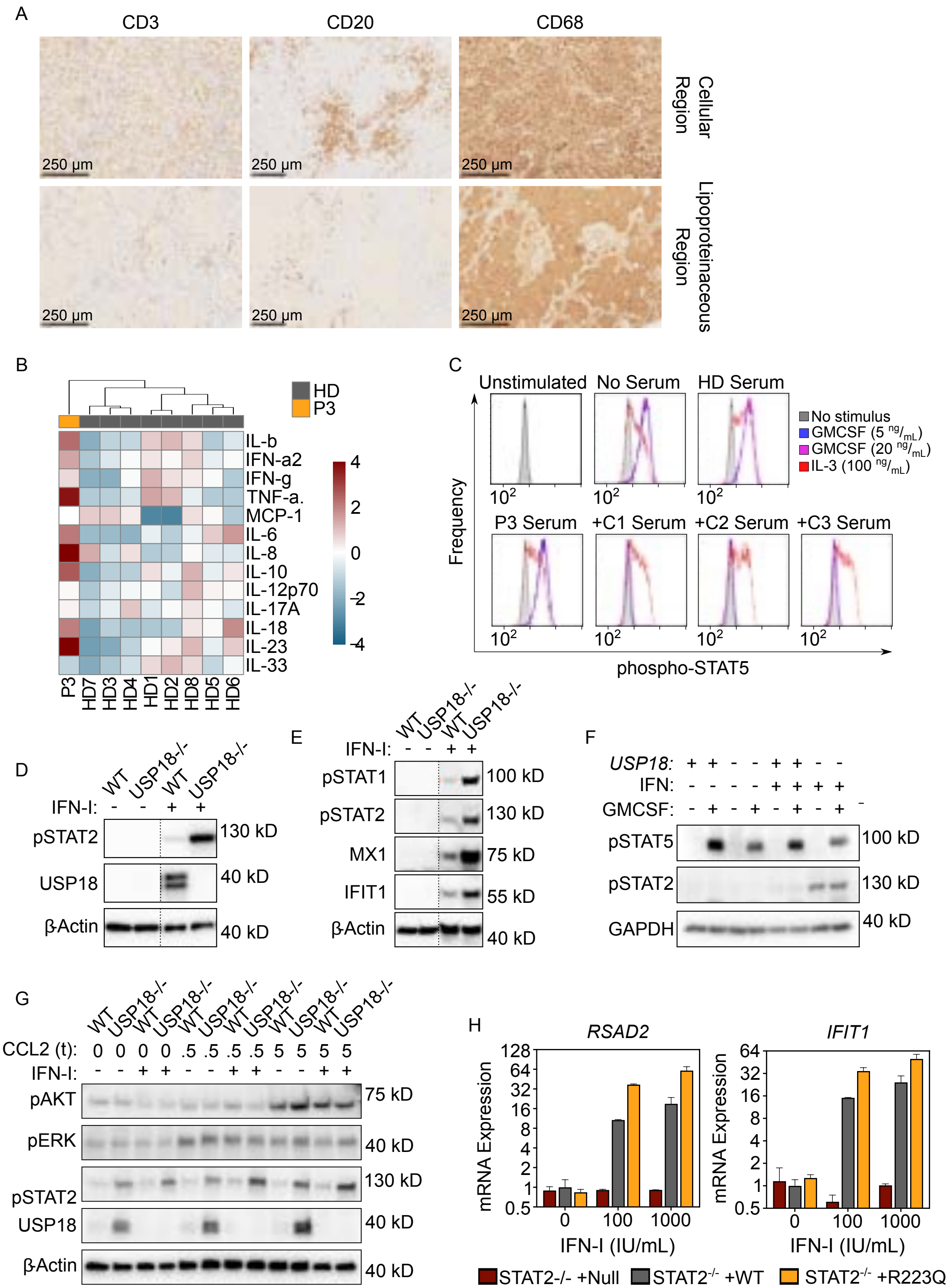
A
CD3
CD20
CD68
Cellular Region
Lipoproteinaceous Region
250 µm
B
HD
P3
IL-b
IFN-a2
IFN-g
TNF-a.
MCP-1
IL-6
IL-8
IL-10
IL-12p70
IL-17A
IL-18
IL-23
IL-33
4
2
0
-2
-4
P3 HD7 HD3 HD4 HD1 HD2 HD8 HD5 HD6
C
Unstimulated
No Serum
HD Serum
P3 Serum
+C1 Serum
+C2 Serum
+C3 Serum
No stimulus
GMCSF (5 ng/mL)
GMCSF (20 ng/mL)
IL-3 (100 ng/mL)
Frequency
phospho-STAT5
10^2
D
WT USP18-/- WT USP18-/-
IFN-I: - - + +
pSTAT2
USP18
β-Actin
130 kD
40 kD
40 kD
E
WT USP18-/- WT USP18-/-
IFN-I: - - + +
pSTAT1
pSTAT2
MX1
IFIT1
β-Actin
100 kD
130 kD
75 kD
55 kD
40 kD
F
USP18: + + - - + + - -
IFN: - - - - + + + +
GMCSF: - + - + - + - +
pSTAT5
pSTAT2
GAPDH
100 kD
130 kD
40 kD
G
WT USP18-/- WT USP18-/- WT USP18-/- WT USP18-/- WT USP18-/- WT USP18-/-
CCL2 (t): 0 0 0 0 .5 .5 .5 .5 5 5 5 5
IFN-I: - - + + - - + + - - + +
pAKT
pERK
pSTAT2
USP18
β-Actin
75 kD
40 kD
130 kD
40 kD
40 kD
H
RSAD2
IFIT1
mRNA Expression
128 64 32 16 8 4 2 1 0.5
0 100 1000
IFN-I (IU/mL)
STAT2-/- +Null
STAT2-/- +WT
STAT2-/- +R223Q
