# Supplementary material for: Familial Pulmonary Alveolar Proteinosis and Type-I Interferonopathy by Mutation of STAT2 (TIMS2)
Source: J Exp Med. Author manuscript; Available in PMC 2026 Aug 11. (PMC7619351; doi:10.1084/jem.20251331)
Supplement: Supplementary file 2 [file EMS216003-supplement-Supplementary_Figures.docx]

**Supplementary Figure 1. Chest CT scans demonstrate pulmonary alveolar proteinosis (PAP)**

(A) Pathognomonic findings of PAP in P1 and P2.
(B) CT scans demonstrating lymphadenopathy.

(C) Evolution of radiologic disease over time across individuals. Patient identifier and their corresponding age indicated on each scan.

**Supplementary Figure 2. MRI imaging of the brain in P1 and P3.**

(A) MRI scans from P1 shown as T2-weighted imaging (left) and diffusion-weighted imaging (right), demonstrating signal abnormalities in the posterior brainstem, midbrain, optic bands and in the sub-thalamic nuclei, as indicated by the red arrows. These abnormalities progress between the ages of 4 months and 1 year especially in the optic and sub-thalamic nuclei.

(B-D) MRI from P3 at age 5 years (B, coronal T2-weighted image demonstrating moderate ventricular dilation), at 6 years following a seizure clinically correlated with right hemispheric signal abnormalities (C, clockwise from top: signal hyperintensity by axial diffusion, decreased apparent diffusion coefficient, and signs of ictal hyperperfusion on arterial spin labelling), and at 6 years 4 months (E, coronal T2-weighted image demonstrating right hemispheric atrophy, right hippocampal atrophy and right ventricular dilation).

**Supplementary Figure 3. Sanger sequencing validation of *STAT2* c.668G>A**

Sanger sequencing performed on a PCR amplicon produced from genomic DNA isolated from whole blood.

**Supplementary Figure 4.** **Basal UPS18 expression in unstimulated B-EBV Cells from P3 and a healthy donor.**

(A) STAT2 and USP18 levels by immunoblotting in EBV-immortalized B lymphoblasts derived from P3 and a healthy control.

(B) STAT1/2 phosphorylation by immunoblotting after IFN-I stimulation at the indicated doses.

Data are representative of at least two independent experiments.

**Supplementary Figure 5. PAP and impaired chemotaxis associate with excessive IFN-I signaling but not GM-CSF defects.**

(A) Immunohistochemistry of two disparate regions of a lung biopsy from P1, a hypercellular area with immune cells filling the airspaces (top) and a region with PAS-positive air spaces lacking significant cellular infiltrate (bottom). Staining performed with antibodies against either CD3, CD20 or CD68 (separate columns), with brown indicating positive signal and a blue counterstain. Scale bars indicate 125 micrometers.

(B) Cytokine levels in plasma from P3 (n=1) or healthy donors (HD1-8, n=8) by Luminex assay. Note healthy donors do not correspond to individuals in Figure 6B.

(C) PBMCs from healthy donors were stimulated with GM-CSF or IL-3 at the indicated concentrations for 15 minutes in the presence of serum isolated from a healthy individual (n=1), P3 (n=1), or patients with known GM-CSF autoantibodies (n=3, labeled +C1, +C2, +C3). Subsequently, cells were assessed for cytokine-induced STAT5 phosphorylation by flow cytometry to measure neutralization capacity in serum. Data represents a single experiment.

(D) Immunoblotting for STAT5 signaling upon brief (15’) GM-CSF stimulation in WT or USP18-/- THP-1 cells.

(E) Immunoblotting demonstrates complete USP18 deficiency in THP-1 cells after CRISPR knockout.

(F) ISG expression and STAT1/2 phosphorylation in THP-1 cells stimulated with IFN-I (100 IU/mL) for 9 hours followed by a 36-hour rest period.

(G) AKT and ERK phosphorylation upon stimulation with CCL-2 (50 ng/ml) for the indicated times (minutes) in THP-1 cells stimulated with IFN-I (100 IU/mL) for 9 hours followed by a 36-hour rest period.

(H) ISG expression in STAT2-reconstituted STAT2-deficient cells after 24 hours of stimulation with IFN-I at the indicated concentrations.

Representative results of at least three independent experiments are shown for each panel, unless otherwise indicated.
